# The Frequency of Rapid Pupil Dilations as a Measure of Linguistic Processing Difficulty

**DOI:** 10.1371/journal.pone.0146194

**Published:** 2016-01-22

**Authors:** Vera Demberg, Asad Sayeed

**Affiliations:** Cluster of Excellence MMCI, Saarland University, Saarbrücken, Germany; University Of Cambridge, UNITED KINGDOM

## Abstract

While it has long been known that the pupil reacts to cognitive load, pupil size has received little attention in cognitive research because of its long latency and the difficulty of separating effects of cognitive load from the light reflex or effects due to eye movements. A novel measure, the Index of Cognitive Activity (ICA), relates cognitive effort to the frequency of small rapid dilations of the pupil. We report here on a total of seven experiments which test whether the ICA reliably indexes linguistically induced cognitive load: three experiments in reading (a manipulation of grammatical gender match / mismatch, an experiment of semantic fit, and an experiment comparing locally ambiguous subject versus object relative clauses, all in German), three dual-task experiments with simultaneous driving and spoken language comprehension (using the same manipulations as in the single-task reading experiments), and a visual world experiment comparing the processing of causal versus concessive discourse markers. These experiments are the first to investigate the effect and time course of the ICA in language processing. All of our experiments support the idea that the ICA indexes linguistic processing difficulty. The effects of our linguistic manipulations on the ICA are consistent for reading and auditory presentation. Furthermore, our experiments show that the ICA allows for usage within a multi-task paradigm. Its robustness with respect to eye movements means that it is a valid measure of processing difficulty for usage within the visual world paradigm, which will allow researchers to assess both visual attention and processing difficulty at the same time, using an eye-tracker. We argue that the ICA is indicative of activity in the locus caeruleus area of the brain stem, which has recently also been linked to P600 effects observed in psycholinguistic EEG experiments.

## Introduction

Pupil size has long been known to reflect arousal [1] and cognitive load in a variety of different tasks such as arithmetic problems [2], digit recall [3], attention [4] as well as language complexity [5–9], grammatical violations, context integration effects [10] and recently even pragmatic effects [11]. All of these studies have looked at the overall effect of pupil dilation; however, raw pupil dilation as a measure of cognitive load is always at risk of confounding the load reflex with the light reflex, especially in settings where the visual surroundings change or where the screen cannot in all conditions be fully controlled for luminosity of all objects on the screen (note also that the light reflex can even pose a problem in constant lighting conditions, because the pupil exhibits irregular oscillation under the influence of constant light). The “Index of Cognitive Activity” or ICA [12–14] proposes to solve this problem by identifying only those rapid dilations which are related to cognitive load but not the light reflex. The ICA separates the effect of the light reflex on pupil size (which causes larger and slower changes in pupil size) from the effect of the load reflex (observable in the frequency of rapid small dilations) by decomposing the raw pupil size signal with different wavelets to obtain high vs. low frequency components of the signal. The ICA is therefore more robust with respect to changes in ambient light than macro-level pupil dilation [12].

Furthermore, the ICA is a highly dynamic measure: it has low auto-correlation at a lag of 100 ms, and almost no correlation with its own value 200ms earlier [15]. Its low degree of autocorrelation makes the ICA potentially suitable for multi-task settings.

If it reliably reflects linguistic manipulations, the ICA could constitute a useful new method to assess processing load using an eye-tracker, in auditory experiments, as well as in naturalistic environments which are not well suited for the use of EEG, e.g. while driving a car. It could therefore usefully complement the range of experimental paradigms currently used. Secondly, if it proves to be robust to with respect to eye movements (which it theoretically should be, because eye-movement related changes would also show up in the signal as slower effects than the load reflex and hence be filtered out during wavelet analysis), the ICA has potential as an additional measure to be used in the visual world paradigm, thus allowing researchers to assess visual attention and cognitive load at the same time.

The goals of the experiments presented in this article are to test whether

- the ICA is sensitive to linguistic manipulations;
- the ICA is robust with respect to fixation position on the screen, making it suitable for use within the visual world paradigm;
- the ICA allows us to tease apart effects of overlapping stimuli, for example in dual task settings.

This article presents the results of three self-paced reading experiments (Expts 1–3) three dual task experiments (driving and language comprehension; Expts 4–6), and one visual world experiment (Expt 7). Results of these experiments are supportive of the ICA being sensitive to linguistic manipulations; we found significant effects in the expected direction in all of our experiments. Furthermore, the experiments show that the ICA is suitable for usage in dual task scenarios and is robust with respect to fixation position, thus making for an ideal additional measure in visual world studies.

But how is it that the pupil muscles can indicate cognitive processing load or indeed linguistically induced processing difficulty? The fact that the light reflex is different from the load reflex and that the two can be disentagled [12] suggests that the ICA probably has little to do with vision, but is rather a non-functional symptom of cognitive load. Recent literature relates pupil dilation to activity in the locus caeruleus (LC) area [16, 17]. [16] report a strong correlation in primates between activity in the locus caeruleus area and pupil size. The locus caeruleus (LC) is a small bilateral region in the brain stem. LC neurons emit the neuro-transmitter norepinephrine (NE), which can be thought of as having an amplifying effect, i.e., neurons will fire at lower levels of excitation and also be inhibited more easily. Norepinephrine therefore facilitates the functional integration of different brain regions, as its enhancing property makes it more easily possible for neurons to synchronize, and thus it is likely to be beneficial in helping processing in the presence of processing difficulty [18]. For a literature review on the LC system, see also [19]. The LC could plausibly affect language processing, because projections from the LC area are very wide-spread in the brain and reach most language related cortical areas [20].

The LC-NE system is also known to affect heart rate and skin conductance, which are known (but slow) indicators of stress as well as cognitive load. NE is thought to have a role in memory retrieval and memory consolidation [18] and has been found to facilitate the functional integration of different brain regions involved in a task. At medium levels of LC-NE activation, this leads to increased performance, while low activation levels happen during drowsiness, and high activation levels lead to distractability. This is consistent with reports on the ICA [12, 14], which observe very low amounts of rapid dilations in a relaxed task, and very high level of rapid dilations during focused concentration on a task.

While the LC area is not an area usually argued to have much of a role in language processing, the LC-P3 hypothesis [21] argues for a tight correlation between activity in the LC region and the P3b component observed in ERP studies as a general reaction to task-relevant stimuli. The P3b component in turn has been proposed to be related to or functionally equivalent to the P600 effect often observed in psycholinguistic experiments [22]. The potential relation between the LC/NE area and the P600 has received further support by a recent paper [23], providing support for this hypothesis by showing in single-trial alignment analyses that P600 latency is more strongly aligned with response times (response times have been argued to be aligned with the P3b [24]), than with stimulus onset. Based on the P600-as-LC/NE-P3 hypothesis [23], we would like to suggest an interpretation of the ICA in which unexpected stimuli or other difficulty with language processing causes the higher cortical areas that are involved with language processing to signal to the brain stem (in particular, to the LC area) that processing resources are needed. The LC area releases norepinephrine, which floods the brain, thereby enhancing information processing in the language processing areas, while also innervating the pupil muscles (and also affecting heart rate, skin conductance etc.), as a side effect. This effect of NE on the pupil muscles may thus be what we measure when we observe more frequent rapid dilations of the pupil as a reaction to our linguistic manipulations.

## The Index of Cognitive Activity

**Fig 1:**
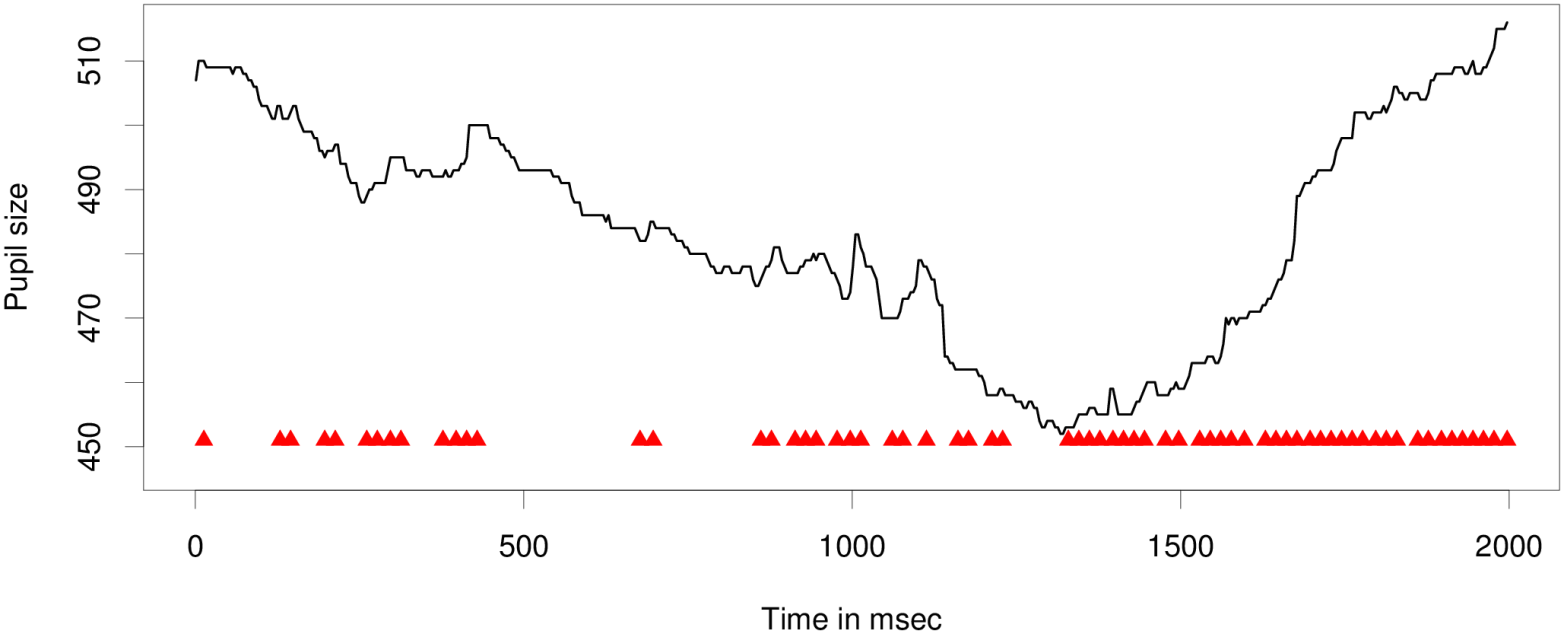
An example of a pupil size recording from our experiment, showing time points of where a rapid dilation was detected according to the ICA software (red triangles).

The Index of Cognitive Activity is a measure of cognitive load which has previously only been evaluated on a small range of tasks [12–14, 25, 26], including digit span tasks, visual tasks, and a simulated driving task. Using the ICA as a measure of processing load is motivated by the finding that pupil size can be affected by two different processes: lighting conditions and cognitive activity. In overall pupil dilation, these two effects are confounded, even when light conditions is stable due to the so-called “light reflex”, meaning that the pupil oscillates irregularly and continually. Pupil dilation is controlled by two groups of muscles: circular muscles, which make the pupil contract, and radial muscles, which make the pupil dilate. Furthermore, we know that the activation and inhibition patterns are different for reactions to light and reactions to cognitive activity [12, 27]: dilations due to cognitive activity are very rapid and small, while changes in pupil size due to lighting are slower and larger. The ICA disentangles these patterns by performing a wavelet analysis on the pupil dilation record to remove all large oscillations and retain only the small and rapid dilations. Among all small dilations and constrictions of the pupil, there are then the ones related to cognitive load in which we are interested as well as some random noise. [12] applies a denoising technique which tests for significance of changes in the signal and sets all non-significant changes to zero. The resulting signal then contains rapid dilations and constrictions larger than the threshold for denoising. For the calculation of the ICA, only the rapid dilations are considered. For an example of a 2-second recording from our own experiment along with marks for where a rapid dilation was detected, see Fig 1.

[14] demonstrates in an experiment that uses a 2x2 design (no task vs. math calculations by light vs. dark room) that the ICA values obtained using this procedure are dependent on the level of cognitive load, but not on light conditions. The ICA has also been related to the task-evoked pupillary response (TEPR), see [28], but has been shown to be more robust with respect to light changes [14].

The method proposed in [12, 14] for calculating the ICA is to count the number of rapid small pupil dilations per second, to normalize by dividing by the number of expected rapid dilations per second, and then to transform the result using the hyperbolic tangent function. The method is patented, and the analysis program, the Cognitive Workload Module, has to be licensed from EyeTracking Inc., San Diego, CA. For details see [12]; results reported here were obtained using EyeWorks 3.8. To obtain a continuous measure, blinks are factored out by linear interpolation of adjacent time spans. By default, an ICA value per second is produced. Existing publications by Marshall refer to averaged tangent-transformed ICA values per second.

For our experiments here, we are interested in a much more fine-grained analysis; in particular, we would like to learn about the time course of the ICA and get an idea of where we should look for a stimulus-related effect. However, existing studies by Marshall and colleagues average ICA values across blocks, i.e., they do not provide any analysis of the more exact time course of the ICA as a response to an experimental manipulation. Furthermore, the compared conditions do not report effect sizes for different levels of difficulty on the same task; rather, they report effect sizes for doing no task vs. solving math problems or doing one task vs. two tasks at the same time. Therefore, we cannot derive from the published studies any specific predictions regarding the expected effect size for linguistic manipulations, or regarding the exact time course of the ICA effect.

The only work that provides a more detailed analysis of the time course of the ICA is [15], who employ the same kind of steering task as the one described here as part of experiment 4. [15] show in a cross-correlation analysis of a steering task and the ICA that the correlation between stimulus and effect on ICA is highest at a time lag of roughly 1.1 seconds. We subsequently conducted a more detailed analysis of this data, and found that subjects who performed well on the task (most subjects) had a latency between stimulus and ICA of 1 second, while subjects who performed very poorly on the driving task showed a delayed or no effect on the ICA (we also replicate this observation in this article as part of experiment 4). As all our participants here are highly proficient (native speakers) of German, we hypothesize that we will be able to observe the effects of our linguistic manipulations also with an approximate lag of 1s. For all of the experiments reported in this article, we report results for a window of 500ms of observations (750–1250ms post stimulus onset). This window was chosen post-hoc, due to the exploratory nature of the current study. From Marshall’s studies [14], we know that we can expect an ICA in the range of 0.67 to 0.87 (which corresponds to 2.4–3.9 rapid dilations per 100ms) for our driving task. For other tasks (e.g., math or visual tasks), Marshall reports much lower ICA values; however, those values are averaged across blocks which don’t require continuous attention. As all of the critical regions of tasks on which we will evaluate require continuous attention, we expect to see ICA values in the range of 2.4 to 3.9 rapid dilations per 100ms across all of our experiments.

In order to provide a more detailed insight into the time course of the ICA, we calculate a per-100-ms ICA value from the number of the rapid dilations per 100ms. Due to the short duration of the 100ms intervals, it does not make sense to interpolate for blinks in this setting, as blinks take about 100ms. We furthermore excluded all data points for which the pupil size estimate was smaller than 2.5 standard deviations from the average pupil size of that participant, because we wanted to be sure to avoid partial blinks. For our analyses here, we will report directly the number of rapid dilations per time span, for ease of interpretation and because the number of rapid dilations are normally distributed, whereas ICA values, due to the hyperbolic tangent transformation, have a skewed distribution; see for more detail also the analysis in Demberg (2013) [15].

All of the data reported in this article was recorded using an EyeLink II eye tracker, at 250Hz on both eyes. The ICA has however also been successfully employed by the developers with other trackers, see [29] for details. The wavelet transformation and calculation of rapid small dilations was performed using the EyeWorks Workload Module software. For each of the experiments, we decided to recruit 24 participants before starting to run the experiment. Each of the experiments also contained 24 items (with the exception of experiment 7, for which we had 20 items).

### Overview

Experiments 1, 2 and 3 directly address our question of whether the ICA reflects *linguistically* induced cognitive load. We chose three well-established experimental manipulations, testing for the effect of ungrammaticality, thematic fit and subject vs. object relative clause effects. Experiments 4–6 test the same stimuli in a dual-task setting where the participants have to do a steering task in a driving simulator and listen to speech-synthesized versions of the stimuli. Experiment 7 tests the processing of short stories containing causal vs. concessive discourse connectives in the visual world paradigm.

## Ethics Statement

The Head of the Saarland University ethics committee confirmed that if no confidential information is collected, if the experiments do not induce a stressful situation and do not involve negative, or emotionally adverse stimuli, then such a study does not need approval to be conducted. All data were anonymized before the authors had access for analysis. Written informed consent was obtained from all participants prior to the start of the experiment. The person to whom the eye image (Striking image) belongs provided consent to have the image of their eye published.

## Experiment 1: Grammatical Gender Mismatch in SPR

### Procedure

We recruited 24 German native speakers as participants in our experiment. Participants were 19–36 years old (average age 24 years), 18 of them were female, and 23 of them were right eye dominant. All of our participants received course credit for their participation.

Materials were presented using the word-by-word self-paced reading paradigm with center-of-screen presentation in order to minimize eye-movements. The materials for the experiment consisted of three training examples as well as 96 German sentences: 24 stimuli each, from experiments 1–3, as well as 24 fillers which were unambiguous relative clauses and contained either semantic or grammatical problems. Half of the items seen by a subject were thus grammatical and plausible stimuli, and half were either ungrammatical or implausible. Each person only saw one version of each item, and we made sure in all experiments that the critical region was not sentence final in order to avoid sentence wrap-up effects in the critical region.

Each sentence was followed by a question asking whether the sentence had been grammatical and made sense. Participants responded using a response pad. Answers were balanced so that “yes” was the correct answer half of the time. Experiment duration was 20–30 minutes.

### Materials

The materials for the gender mismatch experiment included 24 items, where the gender of the determiner and adjective did vs. did not match the grammatical gender of the noun (see Example (1), with the noun in bold face; the full set of items is provided in S1 Text).

(1) Simone hatte eine(n) schreckliche(n) **Traum** und keine Lust zum Weiterschlafen.
  “Simone had a_[*masc*/*fem*]_ horrible_[*masc*/*fem*]_ **dream**_*masc*_ and didn’t feel like sleeping any longer.”

### Data Analysis

We centered and scaled pupil size estimates for each participant. In order to make sure that our results can be attributed to actual changes in pupil size (and not to partial blinks or track loss), we excluded all data points where the pupil size estimate was smaller than 2.5 standard deviations from the average pupil size of that participant, which resulted in a loss of 2% of data points. Binary predictors are encoded using dummy coding.

The data (for this and all other experiments reported in this article) was analysed using R version 3.1.2, lme4 package version 1.1–7 [30]. Spline models were calculated using package gamm4 version 0.2–3 [31].

All of the generalized linear mixed effects models reported in this article include random intercepts under participant and item, as well as random slopes for the predictor of interest (i.e., the linguistic manipulation) under both participant and item, unless otherwise specified (when a model with the full random effect structure did not converge). Confidence intervals were computed using the “Wald” method. We calculated separate models for the ICA on the left eye and on the right eye for a period of 500ms, peaking at 1s post stimulus-onset, as well as regression models that include the data from both eyes in a single regression model. In these models, a random effect of *eye* was included as a nested effect under subject.

The linear mixed effects model used the number of ICA events as a response variable, that is, the number of ICA events for the 500ms target window were added, and values for missing 100ms time windows were interpolated. Each trial is thus represented by one data point per eye. As the “raw ICA” response variable is a count variable, we use a poisson distribution in our mixed effects models. We compared models with and without the linguistic condition as a predictor to see whether our manipulation affected the ICA. As additional predictors in the model, we include the order in which items were shown within in the experiment (as a main effect and as an interaction between grammatical condition and item order), to account for any learning effects during the experiment. Furthermore we tested whether fixation position or the presence of large saccades affected the ICA: we calculated average fixation position in terms of X and Y coordinates, as well as the maximal difference between fixation positions during our critical region. As X and Y axis fixation position may not be linear predictors of the ICA, we always include these factors in our regression models as non-parametric smooth functions using gam / gamm4 models.

We performed backward model selection for fixed effects throughout the experimental analyses reported in this article. Predictors are only included in the final models in case they significantly improve model fit. We additionally calculated models with maximal random effects structure as well as models where we performed forward selection on random effects. All of our tables report results for maximal random effects structure, and the text furthermore reports results from random effects structure as determined by model selection.

### Results

#### Self-paced Reading Times and Question-Answer Accuracy

**Fig 2:**
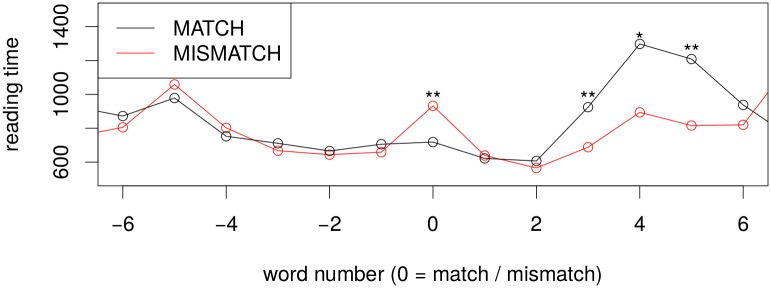
Self-paced reading times for the grammatical gender violation experiment. Critical regions for the experiment is located at word number 0.

As a first sanity check of whether our experimental manipulation was successful, we analysed the self-paced reading times. As expected, reading times were longer on the critical region when the grammatical gender of the noun did not fit the gender marking of the preceding determiner, see Fig 2. A linear mixed effects model with random slopes under subject and item also confirmed that the effect is significant at the critical region: *β* = 176.87, *t* = 2.829, 95%*CI* = [54.32, 299.41]. We also observe that participants read slower in the grammatical condition a few words after the critical region. We interpret this effect as a consequence of the grammaticality judgment task that people had to perform in this experiment: when they had figured out that the sentence is ungrammatical, they read faster because the knew how to answer the question; when the sentence was grammatical, they increase attention and read more slowly as to not miss any problem with the sentence. Question answer accuracies were very high in both conditions for this experiment: 96% correct “yes” answers for the grammatically correct sentences, and also 96% correct “no” answers for the ones containing a gender violation.

Next, we are interested in whether we find a higher frequency of rapid pupil dilations (i.e., higher ICA values) in the critical region of the more difficult linguistic condition.

#### Index of Cognitive Activity

**Fig 3:**
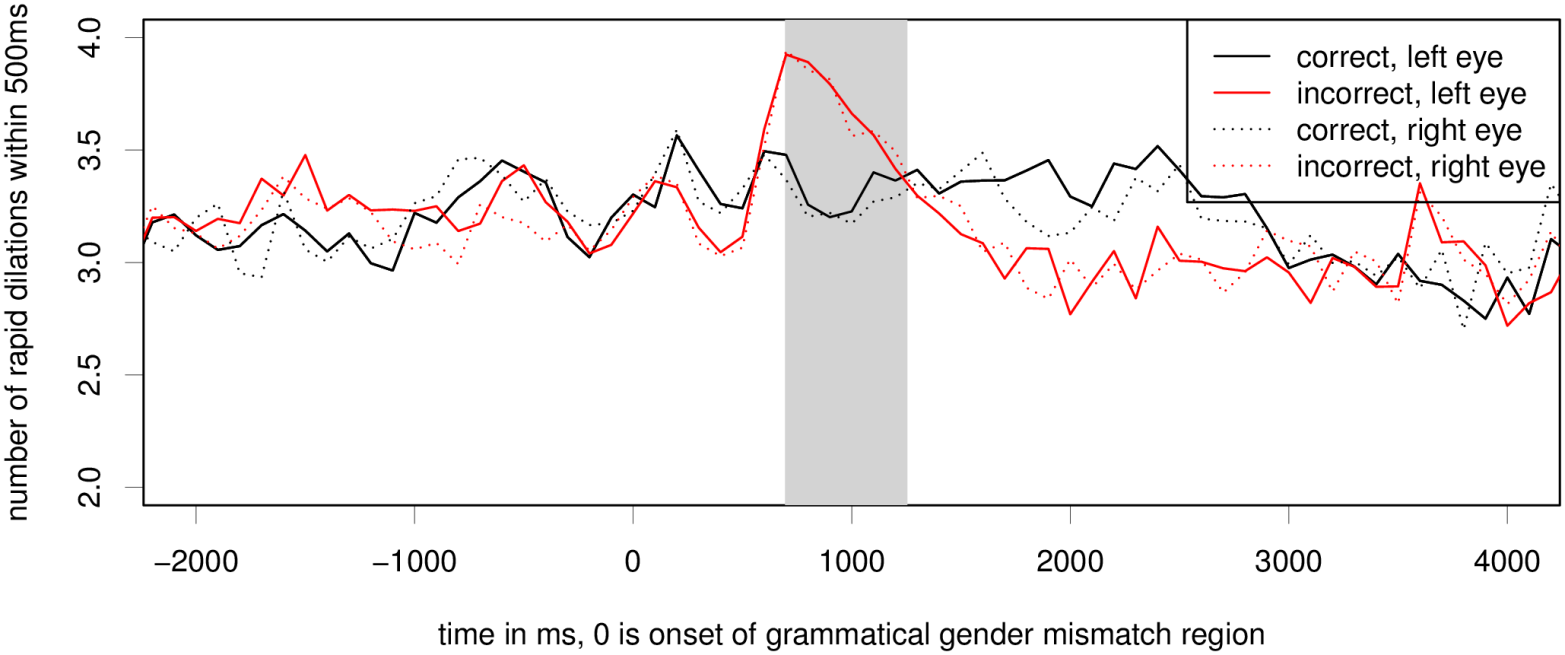
Raw ICA for the grammatical gender violation experiment. Critical region onset for the experiment is located at word number 0; the grey bar marks the hypothesised critical region of 500ms, peaking at 1s. The difference between conditions in this region is significant at *p* < 0.05 on both eyes, see also Table 1.

Fig 3 shows the average number of rapid dilations per 100ms window per time frame. The plotting was performed based on the 100ms bins. We can see a sharp increase in the number of ICA events for the ungrammatical condition in the time period of 600 to 1200 after the onset of the critical word showing on the display. We can also see in the figure that the ICA is very similar for both eyes (compare dotted and solid lines of each color). Another interesting observation from this figure is that the ICA values are lower in the ungrammatical condition compared to the grammatical ones, much like we also observed in self-paced reading times.

Regression analysis showed that the predictors for item order, the interaction of item order and grammaticality, X axis fixation position, and Y axis fixation position did not improve model fit for regression models of either eye for the gender mismatch experiment.

We ran linear mixed effects models for both eyes (separately and combined) to test whether the difference between conditions is statistically significant for a time period of 500ms, peaking at 1s. This region from 750 to 1250ms post critical region onset is marked by a grey bar in Fig 3. In the grammaticality experiment, models with a fixed effect for condition and full random effects structure showed significantly better fit than models without the fixed effect (but still including all the same random effects) (left eye: *χ*^2^ = 4.34, *p* = 0.037; right eye: *χ*^2^ = 5.38, *p* = 0.02; both: *χ*^2^ = 4.66, *p* = 0.031). The resulting model for both eyes is shown in Table 1. We can see that gender mismatch is a significant positive predictor of average ICA values in the region of 750–1250ms post critical region onset. Separate models for each eye yield equivalent results for the predictor gender mismatch (left eye: *β* = 0.133, *z* = 2.34, *p* = 0.19, with maximal random effects structure; right eye: *β* = 0.112, *z* = 2.46, *p* = 0.14, without a slope of gender mismatch under item, as the full random effects structure model didn’t converge).

**Table 1:** Linear mixed effects models for both eyes in the self-paced reading experiment with grammatical gender match / mismatch manipulation.

	ICA			
	*β*	z-val	p-val	95%CI
(Intercept)	2.7649	67.09	<0.00001 ***	[2.684, 2.845]
gender mism	0.1223	2.23	0.026 *	[0.014, 0.230]

RawICA = mismatch +(mismatch | item)+(mismatch | participant/eye)

## Experiment 2: Semantic Anomalies in SPR

### Procedure

Procedure and participants were identical to experiment 1, reported above.

### Materials

We created 24 items that contained a semantic violation such that the selectional restrictions of the verb are violated by one of its arguments and contrasted these with a version of the sentence where the verb was chosen such that the argument is a good thematic role filler for it, as shown in (2); the full set of items is provided in S1 Text.

(2) *Christina schiesst / raucht eine* ***Zigarette*** *nach der Arbeit*.
  “Christina is shooting / smoking a **cigarette** after work.”

### Data Analysis

Data Analysis was identical to the analysis steps for Experiment 1, reported above.

### Results

**Fig 4:**
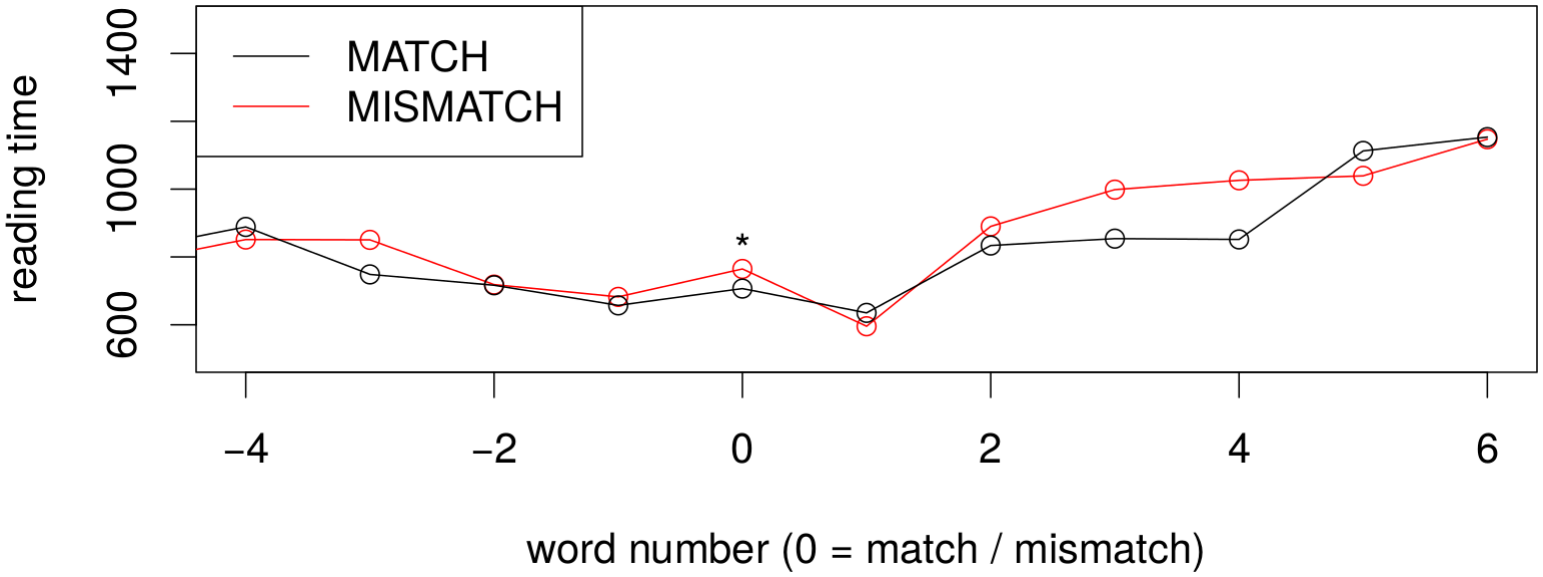
Self-paced reading times for the semantic anomaly experiment. The critical region is located at word number 0.

Fig 4 shows longer reading times on the semantically unexpected word compared to one that fits the context well. Effect size is a bit smaller than the one observed for the gender match/mismatch experiment but is significant at *p* < 0.05 according to a linear mixed effects model including slopes for semantic match/mismatch under both item and participant (*β* = 57, t-val = 2.149, 95%CI = [7.20, 110.54]). There is also a slowdown a few words after the critical region, however, this later difference between conditions is not statistically significant.

#### Self-paced Reading Times and Question Answer Accuracy

Question answer accuracies for this experiment were again very high: 99% correct (“yes” answers) in the congruent condition and 96% accuracy (“no” answers) in the semantic violation condition.

#### Index of Cognitive Activity

**Fig 5:**
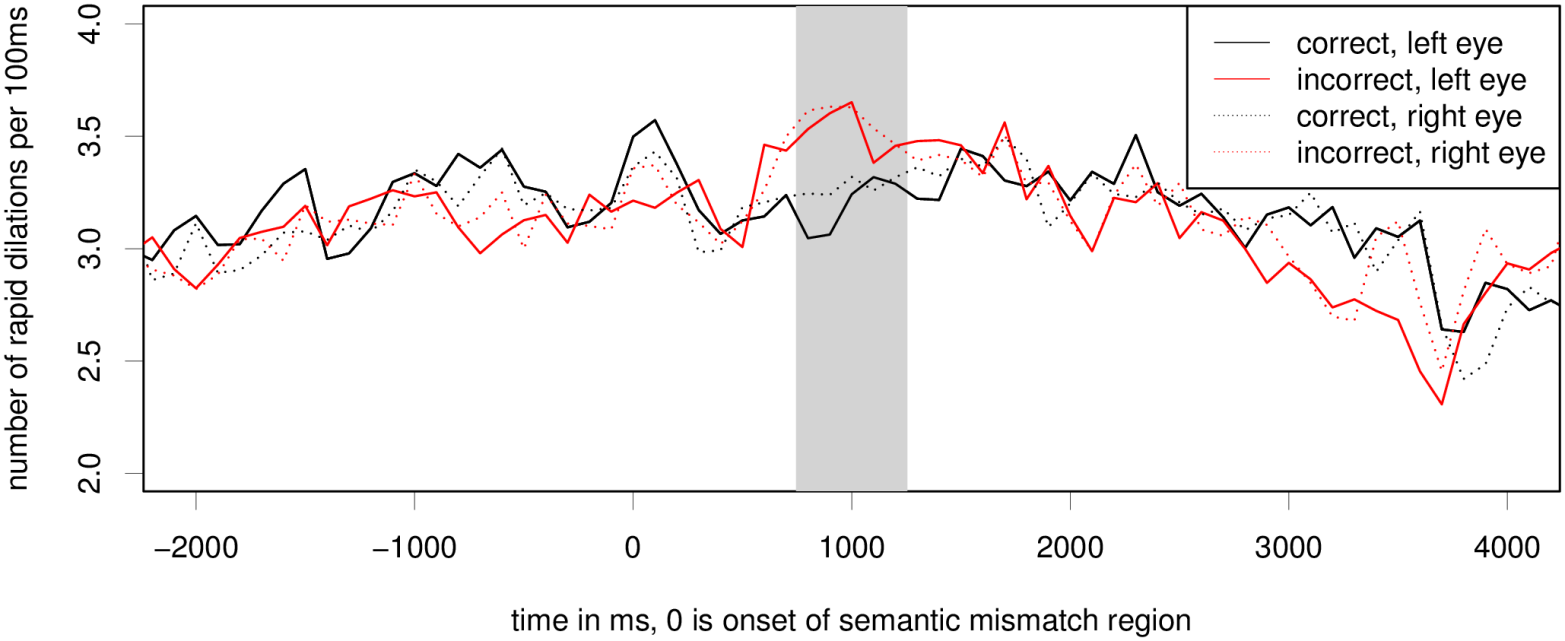
Raw ICA for the semantic fit experiment. Critical region onset for the experiment is located at word number 0; the grey bar marks the region of 500ms, peaking at 1s. The difference between conditions is significant at *p* < 0.001 for the left eye and *p* < 0.01 for the right eye, see also Table 2.

Fig 5 shows the average number of rapid dilations per 100ms window per time frame. We can see a clear increase of the number of ICA events for the ungrammatical condition in the time period of 600 to 1100ms after the onset of the critical word. Again, the ICA effect is similar for both eyes.

We again test linear mixed effects models for both eyes separately as well as a model with both eyes included, for the period of 750–1250ms after onset of the critical word. Backward model selection shows that fixation position for x and y axis in the experiment are not significant predictors of the ICA in the semantic anomaly experiment for either eye, however, the interaction between item order and condition is significant on the right eye and in a model including both eyes. For this dataset, a linear mixed effects model with a random slope under both item and subject does not converge when run on each eye separately. Model comparison shows that a model with no random slopes for semantic violation is not significantly different from one with random slopes under subject or from one with random slopes under item. A model including a fixed effect for condition and maximal converging random effects structure (including a random slope for semantic violation under subject) is better than a model with random slopes only, on both eyes (left eye: *χ*^2^ = 11.66, *p* = 0.0006; right eye: *χ*^2^ = 10.73, *p* = 0.0010); both eyes: *χ*^2^ = 8.1104, *p* = 0.0025. Table 2 reports modelling results for the maximal random effects structure that converges. The estimates for semantic mismatch in a model including only the left eye with maximal converging random effects structure is *β* = 0.108, *z* = 3.85, *p* = 0.00012. In a model including only the right eye, the estimate for semantic mismatch is *β* = 0.094, *z* = 3.61, *p* = 0.00030.

**Table 2:** Linear mixed effects models for semantic anomaly in SPR.

	ICA			
	*β*	z-val	p-val	95%CI
(Intercept)	2.7540	71.13	<0.00001 ***	[2.678, 2.829]
gender mism	0.0981	2.87	0.0042 **	[0.031, 0.165]
itemOrder	0.0035	0.18	0.857	[−0.034, 0.041]

RawICA = mismatch + (mismatch | item)+(mismatch | participant)+(1 | participant/eye)

## Experiment 3: Relative Clauses in SPR

### Procedure

Procedure and participants were identical to Experiments 1 and 2, reported above.

### Materials

We used German locally ambiguous subject relative clauses (SRC) vs. object relative clauses (ORC) based on the materials by [32] (some of the sentences were modified and some were replaced), see Example (3); the full set of our items is provided in S1 Text. The object relative clause is known to be harder to process than the subject relative clause, see e.g., [32].

(3) *Die Nachbarin, [* ***die***_*sgnom*/*acc*_ *einige*_*pl nom/acc*_ *der Mieter auf Schadensersatz verklagt **hat***_*sg*_/ **haben***_pl_]*_*RC*_, *traf sich gestern mit Angelika*.
  “The neighbor, [whom some of the tenants sued for damages / who sued some of the tenants for damages]_*RC*_, met Angelika yesterday.”

When reading such a sentence, people will usually interpret the relative pronoun *die* as the subject of the relative clause and the following noun phrase *einige der Mieter* as the object. This interpretation is compatible with the embedded singular-marked verb hat at the end of the relative clause. Encountering the verb *haben*, which has plural marking, leads to processing difficulty: in order to make sense of the relative clause, readers need to reinterpret the relative pronoun *die* as the object of the relative clause and the following noun phrase *einige der Mieter* as its subject. (Note that the sentences are all grammatical, as the relative pronoun and following NPs are chosen such that they are ambiguous between nominative and accusative case marking.)

### Data Analysis

Data Analysis was identical to the analysis steps for Experiment 1, reported above.

### Results

#### Self-paced Reading Times and Question Answer Accuracy

**Fig 6:**
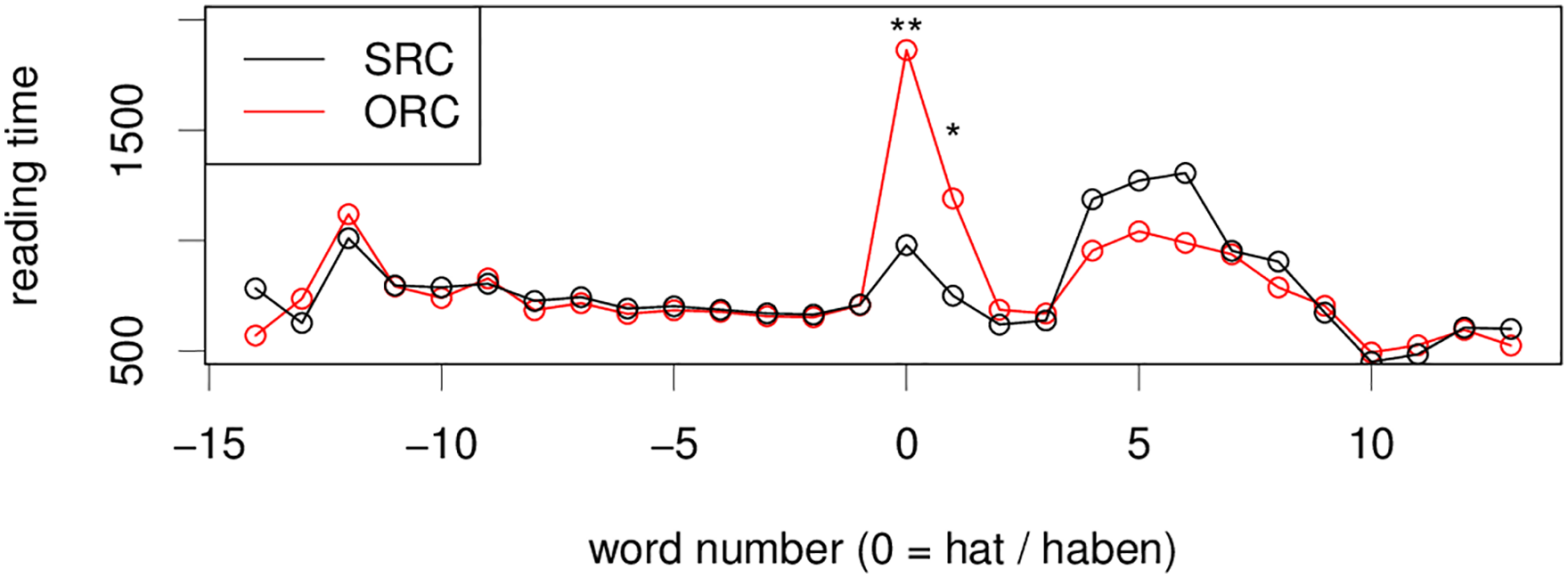
Self-paced reading times for the RC Experiment. Critical region *hat/haben* is located at word number 0. Extremely slow reading times in the ORC case are reflective of the high difficulty in this condition.

Self-paced reading times for the relative clause experiment show a very strong effect of relative clause condition: the critical region is read significantly slower in the object relative clause than in the subject relative clause (*β* = 463.3, *t* = 2.466, 95%*CI* = [44.5, 863.4]) in a linear mixed effects model with full random effects structure, see also Fig 6. Effect size is much larger for this experiment than for the previous two, indicating that participants had interpretation difficulties. We also see a spillover effect on the next word.

Question answer accuracies confirm that the object relative clauses were difficult, sometimes to the point of garden-pathing: while 92.3% of questions following the subject relative clause were correctly answered with “yes”, only 63.8% of object relative clauses were correctly answered with “yes”. More detailed analysis also shows that there are large differences among participants and that the error rate went down during the course of the experiment; about 75% of participants consistently answered the grammaticality judgment questions for ORCs correctly by the end of the experiment. Two participants consistently kept rating the object relative clause as ungrammatical throughout the experiment.

#### Index of Cognitive Activity

**Fig 7:**
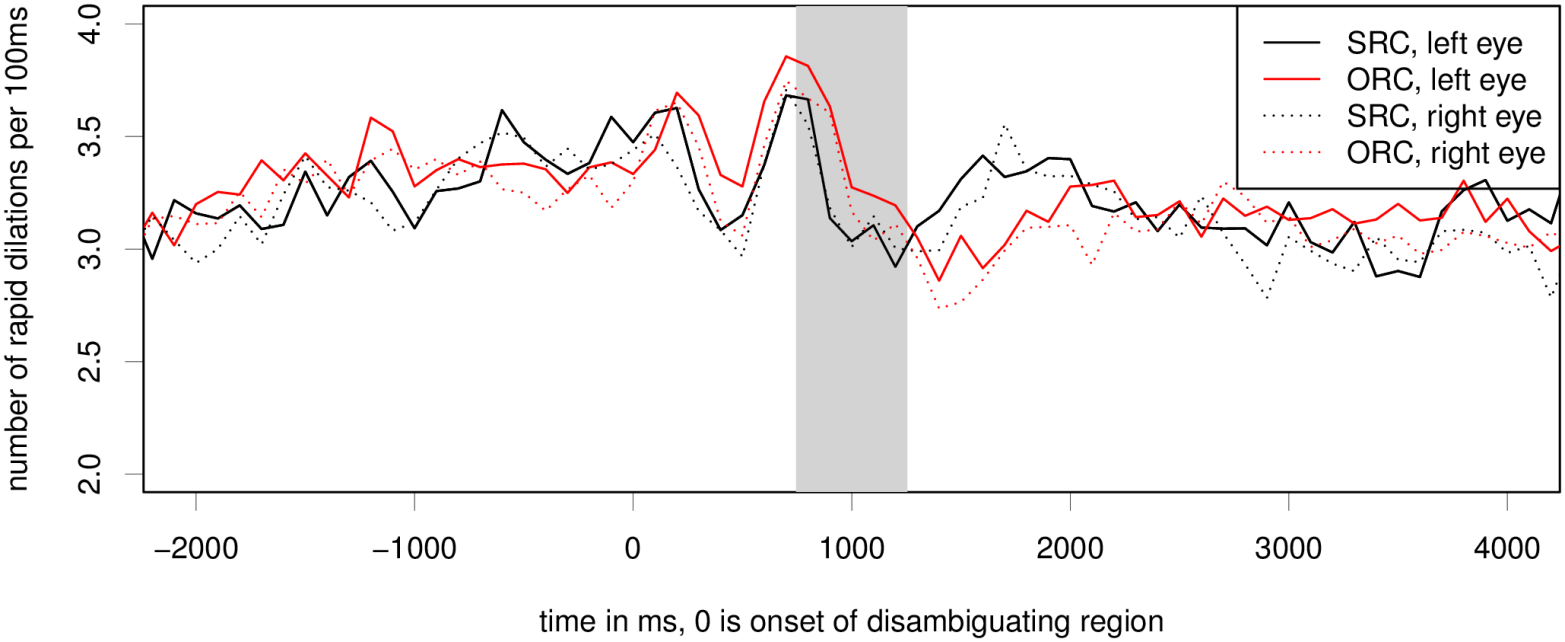
Raw ICA for the relative clause experiment. Critical region onset for the experiment is located at word number 0; the grey bar marks the hypothesized critical region of 500ms, peaking at 1s. For details, see Table 3.

Fig 7 shows a smaller difference between conditions in this experiment than for experiments 1 and 2. However, we can see that the number of rapid dilations is consistently higher for the object relative clause than for the subject relative clause in the critical region. Furthermore, there is a large peak in ICA for both eyes in both conditions in the time period from 500 to 1000ms after the onset of the disambiguating region. Like in experiment 1, there is also an later time period (1300–2000ms after the critical region onset) during which the number of rapid dilations for the simpler SRC is higher than for the more difficult ORC conditions. Note that in the case of this experiment, during the time period of 1300–2000ms after the critical word, participants have on average already moved on to the next word in the subject relative condition, while they are still looking at the critical region in the object relative condition; compare reading times in Fig 6.

We again test linear mixed effects models for both eyes for the period of 750–1250ms after onset of the critical word. Backward model selection shows that the interaction of item order and relative clause type significantly improves model fit on both eyes. The coefficient for the interaction is negative; this means that the effect of relative clause type is large at the beginning and gets smaller during the course of the experiment. The significant interaction between RC type and item order thus indicates a learning effect—as participants have observed more object relative clauses, ORCs may become more expected and easier to process. Furthermore, there were a number of large eye movements along the y axis during the critical region of the relative clause stimuli. Our predictor for eye movements along the y axis was also a significant predictor of the ICA on both eyes. For the left eye model and the model including both eyes, removing relative clause condition as a main effect significantly decreases model fit (left eye: *χ*^2^ = 3.88, *p* = 0.048; right eye: *χ*^2^ = 0.285, *p* = 0.593; both: *χ*^2^ = 4.3738, *p* = 0.0365). On the left eye, the effect of the object relative clause is *β* = 0.099, *z* = 2.48, *p* = 0.013, while the effect of the interaction of ORC and item order is *β* = −0.049, *z* = −2.36, *p* = 0.018. For the right eye, we find no significant effect of RC type (*β* = 0.042, *z* = 0.73, *p* = 0.46); but a significant interaction of RC type and item order (*β* = −0.075, *z* = −3.47, *p* = 0.0005).

In a model including both eyes, the effect of relative clause type does not reach significance (*β* = 0.071, *z* = 1.46, *p* = 0.14), but we again find a highly significant interaction of RC type and item order (*β* = −0.062, *z* = −4.07, *p* < 0.0001). In order to confirm whether we are observing a learning effect in the ICA effect (which would also be consistent with results from the behavioural measures reported above), we run a model including only the first half of the experiment. This analysis confirms that the first half of the experiment does show a main effect of relative clause type, with object relative clauses leading to higher ICA than subject relative clauses, see Table 3 for full results.

**Table 3:** Linear mixed effects models for ICA in SPR relative clause experiment. Results for data from first half of experiment.

	ICA			
	*β*	z-val	p-val	95%CI
(Intercept)	2.728	53.48	<0.00001 ***	[2.685, 2.828]
object RC	0.108	2.09	0.036 *	[0.007, 0.210]
movement Y axis	−0.041	−2.93	0.003 **	[−0.068, −0.013]

RawICA = RC + Y move+ (RC| item) +(RC| participant)+(1| participant:eye)

Adaptation or learning effects are known to occur during psycholinguistic experiments. The present experiment hence shows that the ICA reflects the effects of linguistic processing difficulty incurred by participants when first encountering object relative clauses, and in addition is also reflective of participants’ adaptation during the course of the experiment, in which object relative clauses become easier to process (as also evident by the increased number of correct responses to the grammaticality judgment question).

On the right eye, there is evidence for a learning effect: the number of rapid dilations in the critical region is higher in the object relative clause condition during the first half of the experiment, but the effect disappears (for most subjects) during the second half of the experiment. Therefore, a main effect of relative clause type does not reach significance when removing the interaction with item order. A similar tendency for a learning effect exists for the left eye (even if it did not reach significance there).

## Overall Discussion for ICA in reading experiments

We found higher rates of rapid pupil dilations in the critical region of 750 to 1250 ms after stimulus onset for the more difficult linguistic condition in all three experiments. Effects were similar for both eyes. Taken together these first results provide strong initial evidence that the ICA measure is sensitive to linguistically induced processing difficulty.

A few observations made during the self-paced reading experiments deserve more discussion. In the relative clause experiment we find a peak in rapid dilations for both eyes in the time period of 500 to 900ms after the appearance of the critical word. We interpret this peak as increased attention at the critical region for this word: As the self-paced reading data and the question answering data reveal that participants had trouble with the object relative clauses, and showed a learning effect during the course of the experiment, we think that participants learned to increase attention for the critical region of the relative clauses, which is reflected in the increased number of rapid dilations in both conditions of the RC experiment.

Furthermore, we saw that a peak in the ICA for the critical condition was followed by a relative decrease in the ICA in a slightly later time period. We see two possible explanations for this effect: one could be that this effect is task-dependent, i.e., due to asking whether the sentence was grammatically correct and made sense, and might hence reflect a decrease in attention after the violation was detected. We would hence predict this effect to disappear when using a different task as comprehension questions. Alternatively, it might be that a large effect leads not only to a high ICA effect but also to very large overall pupil dilation, which is then followed by a constriction of the pupil, during which no, or only very few, ICA events are observed.

While there are some differences between participants in terms of the strength of the ICA effect as a function of linguistic condition, we found the overall reaction to be quite consistent: in each of the three experiments, > 80% of participants showed the expected effect of linguistic condition on the ICA.

## Experiment 4: Gender mismatch in dual task driving and listening

The first three experiments have shown that the ICA is sensitive to linguistic manipulations. Experiments 4 to 6 test whether the effects can be replicated in a dual task setting in which a driving task is performed simultaneously with a language comprehension experiment. We know from previous related work [15, 26] that the ICA is also sensitive to the driving task. We here attempt to replicate earlier results in terms of the latency between the ICA and the steering events, and analyze the effect of steering on the ICA in order to adequately account for both effects in our analyses.

### Procedure, Task, and Methods

We recruited 24 participants. Data was lost for one of the participants before analysis, resulting in valid data from 23 participants. Out of these, 13 were females. Almost all of them were right eye dominant (22), and they were aged between 19 and 32 years, with a mean age of 24 years. All participants were native speakers of German, and were paid for their participation in the experiment. None of the participants had taken part in the self-paced reading experiment or an earlier version of a driving simulation experiment.

Our simulated driving task, the OpenDS ConTRe task [33], is derived from two well-known psychological tasks used in numerous dual-task experiments: 1) the tracking task (also “perceptuomotor tracking task” or “pursuit tracking task”) and 2) the reaction task [34]. In the experiments reported in this article, only the tracking portion of the ConTRe task was used. Tracking is realized as steering in ConTRe. Turning the steering wheel influences the lateral position of the vehicle/viewpoint. The ‘steering bar’ (blue vertical bar) is always in the center of the screen. A second vertical bar, the ‘reference bar’, is colored yellow and moves autonomously to random lateral positions on the road, stopping there for two seconds before moving to a different location. Lateral movement of both bars is limited by the solid side markings, preventing the driver from leaving the road. The movements of the reference bar are unpredictable from the point of view of the driver. The driver’s task is to control the lateral position of the steering bar via the steering wheel, such that the two bars overlap as much as possible.

The perspective of the subject is from the simulated car, which moves autonomously at a constant speed along a straight road consisting of two lanes per direction. The simulated car can only drive on the lanes in the correct direction, and it is also not possible to accidentally drive onto the grass beside the road (to avoid stress to the participant for having an accident). A figure showing the ConTRe task used in our experiment is provided in the supplementary information S1 Fig; a demo video of the task is also available at http://www.opends.de/software/driving-tasks/15-controlled-driving-tasks/87-contre-task. The ConTRe steering task is highly continuous and allows for manipulation of task difficulty at run time. The difficulty of the steering task depends on the speed of the reference bar as well as the difference in maximum speed between the reference bar and the steering bar (if the steering bar has a high maximum speed, participants can more quickly catch up with the reference bar). For this experiment, we created two difficulty settings (easy and difficult; for parameters, see Table 4).

**Table 4:** Difficulty settings used for the ConTRe steering task in our experiments.

	easy	difficult
speed of reference bar (m/sec)	1.0	2.5
max. speed of steering bar (m/sec)	2.0	4.0
longitudinal speed (km/h)	40	70

Our experimental setup included a screen in combination with a gaming steering wheel (Logitech Driving Force GT), which was attached to the table. During the experiment, drift correction for the eye-tracker was done by the experimenter whenever required (without interrupting the driving simulation; this is possible as the visual target on the screen does not move vertically).

After reading instructions, participants were trained in the driving task for around three minutes. The experiment consisted of four blocks in randomized order, two blocks of difficult driving and two blocks of driving in the easy condition. Within each block there were two minutes of single task driving and four minutes of dual tasking.

### Materials

Materials were identical to the materials used in experiment 1, consisting of 24 sentences which manipulated grammatical gender to be either consistent or inconsistent with the previous determiner and adjective, as well as 72 fillers (24 of which were items of experiment 5, 24 from experiment 6 and 24 additional fillers). All materials were in German.

In the present dual task experiment, these stimuli were speech-synthesized using the MARY speech synthesizer [35], and then audited and corrected for intelligibility. We used synthesized speech (as opposed to recorded natural speech) in order to avoid confounds of prosody and to be more similar to real dual task applications of interaction with an automatic dialog system while driving.

### Data Analysis

**Fig 8:**
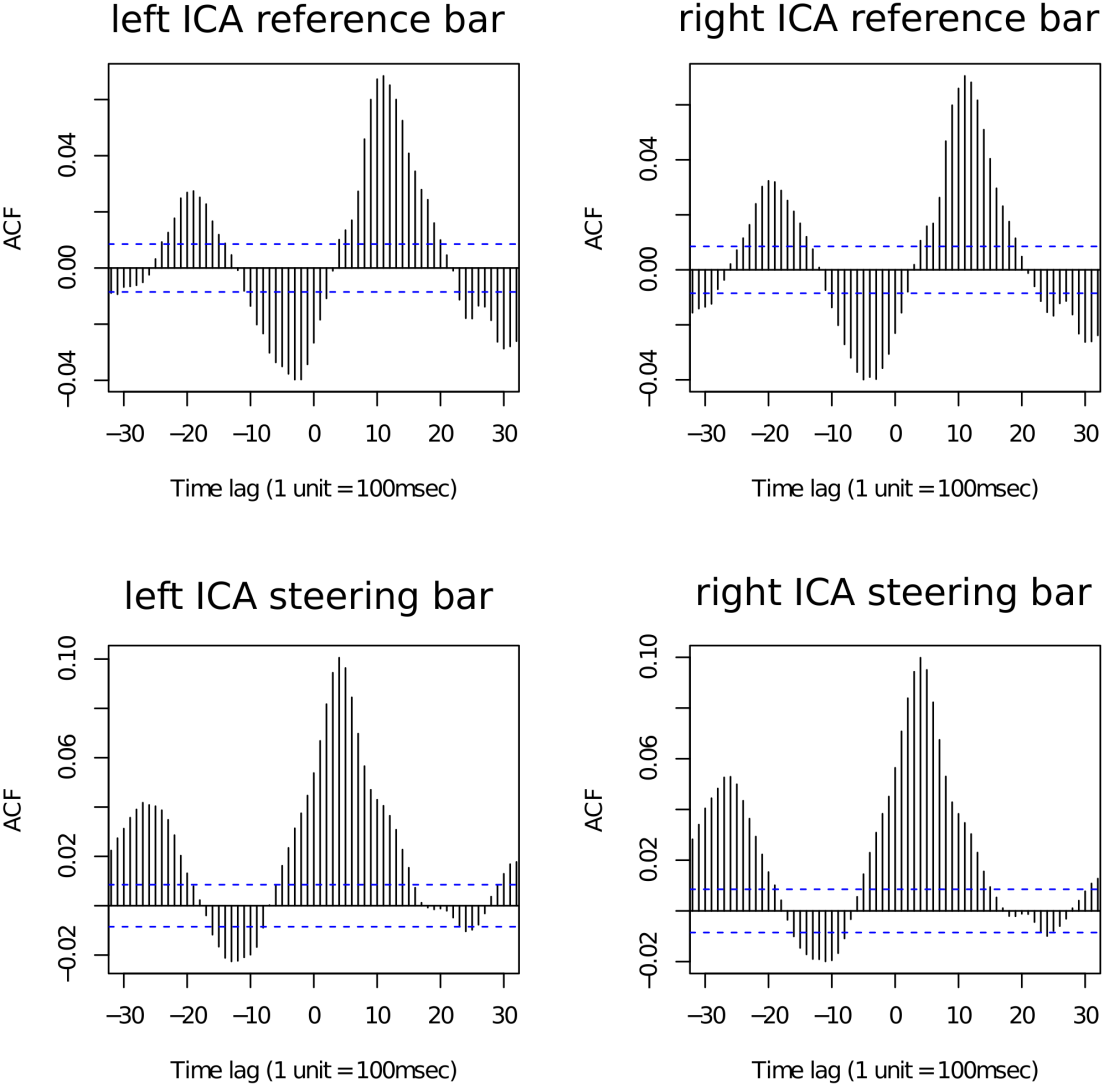
Cross-correlations for the ICA with the reference bar and the steering bar, respectively. Cross-correlation values for the reference bar (top plots) and steering bar (bottom plots) with the ICA of the left eye (left plots) and right eye (right plots). Dashed lines indicate 95%CIs. The periodicity in the cross-correlation plot is due to the periodicity of the steering task. We can see that the correlation of the ICA is higher for steering (i.e. the driver’s own reaction) than for the reference bar (i.e., the stimulus).

In order to evaluate the latency of the effect of the driving task on the ICA, we calculated cross-correlations between these time series using the R mgcv package version 1.7–29 [36]. We found a significant positive cross-correlation between reference bar speed and the ICA on both eyes, starting at a time lag of about 400ms and peaking at a lag of about 1.1 seconds (top plots of Fig 8). Consistent with data from an earlier experiment [15], the more detailed per-subject analysis showed that subjects can be separated into ones who perform very well on the driving task and ones who steered less well. For the ones with good performance, the correlation peaks at 1 second delay. The periodicity seen in the graphs is due to the periodicity of the steering task. The bottom two plots of Fig 8 show a significant cross-correlation between the speed of the subject-controlled steering bar and the ICA. The dashed lines indicate that correlations outside these lines are significantly different from zero at confidence of 95%. The correlation between the ICA and the steering bar is larger than the correlation between the ICA and the reference bar. Given that the steering movement is a reaction to the movement of the reference bar, the latency of the ICA with respect to the steering bar is much smaller (peaking at about 400ms offset).

Fig 8 shows only the effect in the difficult driving condition, but the same pattern also holds for the easy driving setting, as well as in the dual task condition, and constitutes a successful replication of [15]).

For all regression models with the ICA as a dependent variable, we also included steering bar speed as a predictor of the ICA. Based on the results of the single-task driving periods of the experiment, we shifted the steering bar speed data by 400ms, the time lag at which the cross-correlation of steering bar speed and the ICA peaked. Time-shifting steering bar speed improves model fit. Steering velocity and linguistic condition were never significantly correlated. We follow the analysis methods explained as part of experiment 1, and report results for the ICA without hyperbolic tangent transformation. The models we report first regress the steering speed against the ICA for the 100ms windows as well as any effects of fixation position on the screen and subsequently calculate the effect of the linguistic manipulation based for the average residuals of this model over a 500ms time window peaking at 1s post critical word onset.

We again include X and Y axis fixation position, item order, and the interaction between item order and linguistic condition as additional potential predictors and perform backward model selection. Note that in this task, again, fixations are almost always directed to the middle of the screen, as the user-controlled bar is always in the middle of the screen (the road moves under the car and is not always centered with respect to the screen), whereas the randomly moving target bar moves along the x axis, but only does so over the width of the road. Participants mostly fixate on the steering bar in order to achieve overlap with the target bar.

### Results

#### Question Answering Accuracies

Question answering accuracies were less high for the spoken experiment than for the self-paced reading one for the gender match/mismatch materials: While in the self-paced reading experiment, accuracies were at 96% correct for both match and mismatch conditions, they are at 93.1% correct “yes” answers for the match condition, and 85.5% correct “no” answers for the mismatch condition. We attribute this difference (in particular, the one for the mismatch condition) to mishearing of the synthesized speech stimuli, i.e., people potentially didn’t perceive the error, or attributed the deliberate wrong gender to a problem with speech synthesis as opposed to an ungrammaticality in the sentence.

#### Index of Cognitive Activity

**Fig 9:**
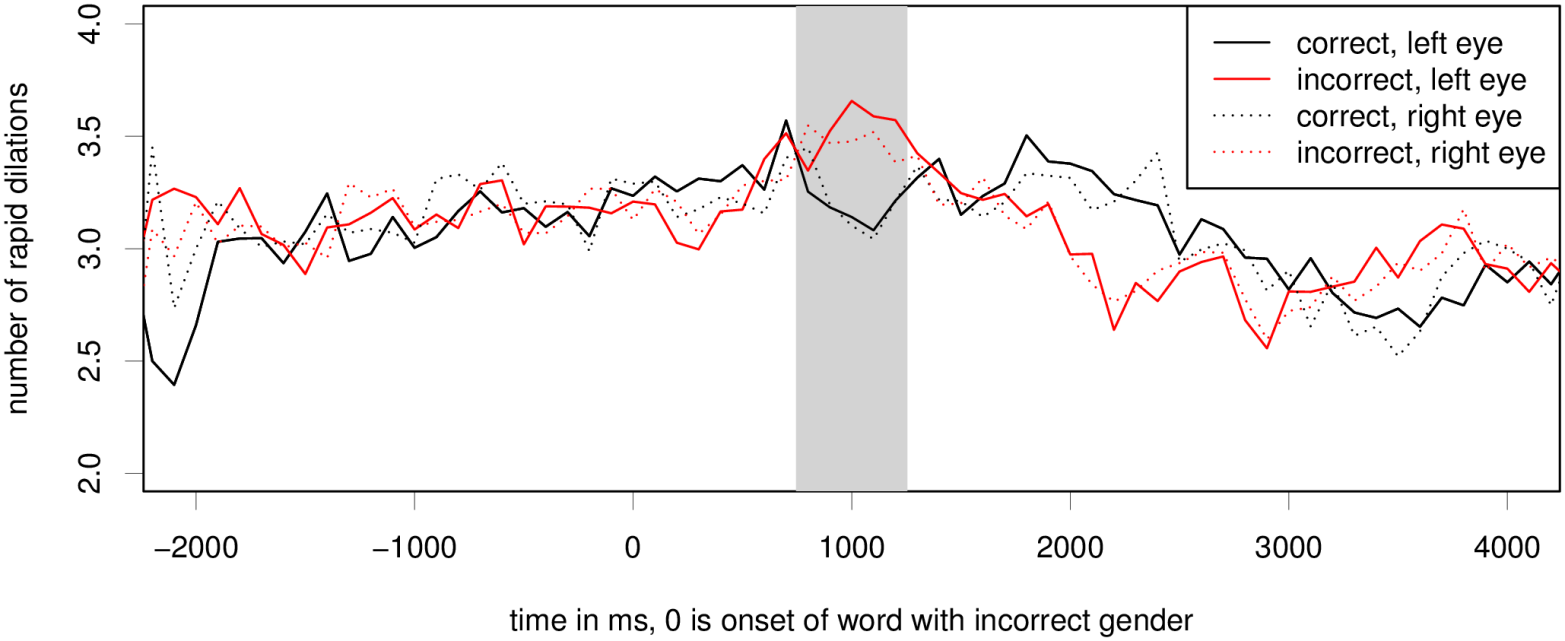
Raw ICA for the dual-task grammatical gender violation experiment. Critical region onset for the experiment is located at word number 0; the grey bar marks the region of 500ms, peaking at 1s. The difference between conditions was statistically significant, see also Table 5.

Fig 9 shows that there were more rapid pupil dilations for the grammatical gender mismatch condition compared to the condition with correct grammar. Just like in experiment 1, the relative increase of rapid dilations in the critical region is followed by a relative decrease of rapid dilations in this condition. Just as in the single-task experiments, effects appear again very similar for both eyes.

Backward model selection revealed that including the predictors for item order and the interaction of item order and condition did not significantly improve model fit. On the other hand, we found a significant positive effect of gender mismatch as well as steering movements on the ICA. Following our earlier findings, the steering movement was shifted by 400ms with respect to the ICA. We then averaged steering bar speed with respect to the 500ms target period and included it as a predictor in the model. We found a significant effect of steering bar movement on the ICA (a model with steering bar movement as an additional random slope under subject did not converge). The dual task experiments contains more eye movements than the (center-presentation) self-paced reading experiments, due to the nature of the task. We therefore don’t exclude data with eye movements, but account for them in the regression analysis. X and Y fixation position (when added as a smooth function to the model) did also not have a positive effect on the ICA. However, we found small significant effects of large eye movements (i.e., large shifts in X and Y position during the critical region). We therefore included these effects in our model. Including these predictors did not affect results on the predictors for linguistic condition and driving task. Results for a model including the data from both eyes is provided in Table 5. Including gender mismatch as a main effect improved model fit significantly (*χ*^2^ = 7.22, *p* = 0.0072).

**Table 5:** Linear mixed effects models for left and right eye in dual task gender match/mismatch.

	ICA			
	*β*	z-val	p-val	95%CI
(Intercept)	2.757832	96.26	< 0.00001 ***	[2.701, 2.813]
gend mismatch	0.088657	2.91	0.00357 **	[0.029, 0.148]
steering	0.036270	4.61	< 0.00001 ***	[0.021, 0.051]
movement X axis	0.025162	2.80	0.00510 **	[0.007, 0.042]
movement Y axis	−0.025430	−2.78	0.00536 **	[−0.043, −0.007]

RawICA = gend mism + steering+ X move + Y move +(gend mism| item) +(gend mism| participant)+(1| participant:eye)

We also ran analyses for each eye separately. Gender mismatch was a positive significant predictor in a maximal random effects model for the left eye (*β* = 0.10268, *p* < 0.0001), and for the right eye (*β* = 0.0732, *p* = 0.019). Both models also included steering bar speed as a significant positive predictor, and the model for the right eye also includes item order as a significant negative predictor.

## Experiment 5: Semantic anomalies in dual task driving and listening

### Procedure

Procedure was identical to the one described for experiment 4.

### Materials

Materials for this experiment were identical to materials used for experiment 2, and speech-synthesized using the MARY synthesizer, as in experiment 4. For the analysis of our semantic anomaly experiment, we had to exclude three items, because the spoken word durations for the critical words (*Organteile*, *Rechtsanwalt*, *Prüfungsergebnis*) in these three items were much longer than those for the other items in the experiment, which means that expected post-stimulus reaction times are not comparable. These words also exhibited particularly long reading times in the self-paced reading experiment.

### Data Analysis

Data analysis was done in the same way as described for experiment 4.

### Results

#### Question Answering Accuracies

Question answering accuracies were very similar in the present dual task experiment to the single task self-paced reading experiment using the same stimuli: for the congruent sentences, 97.3% of responses were correct “yes” responses, and for the ones with a semantic anomaly, 96.3% of answers were correct “no” responses.

#### Index of Cognitive Activity

**Fig 10:**
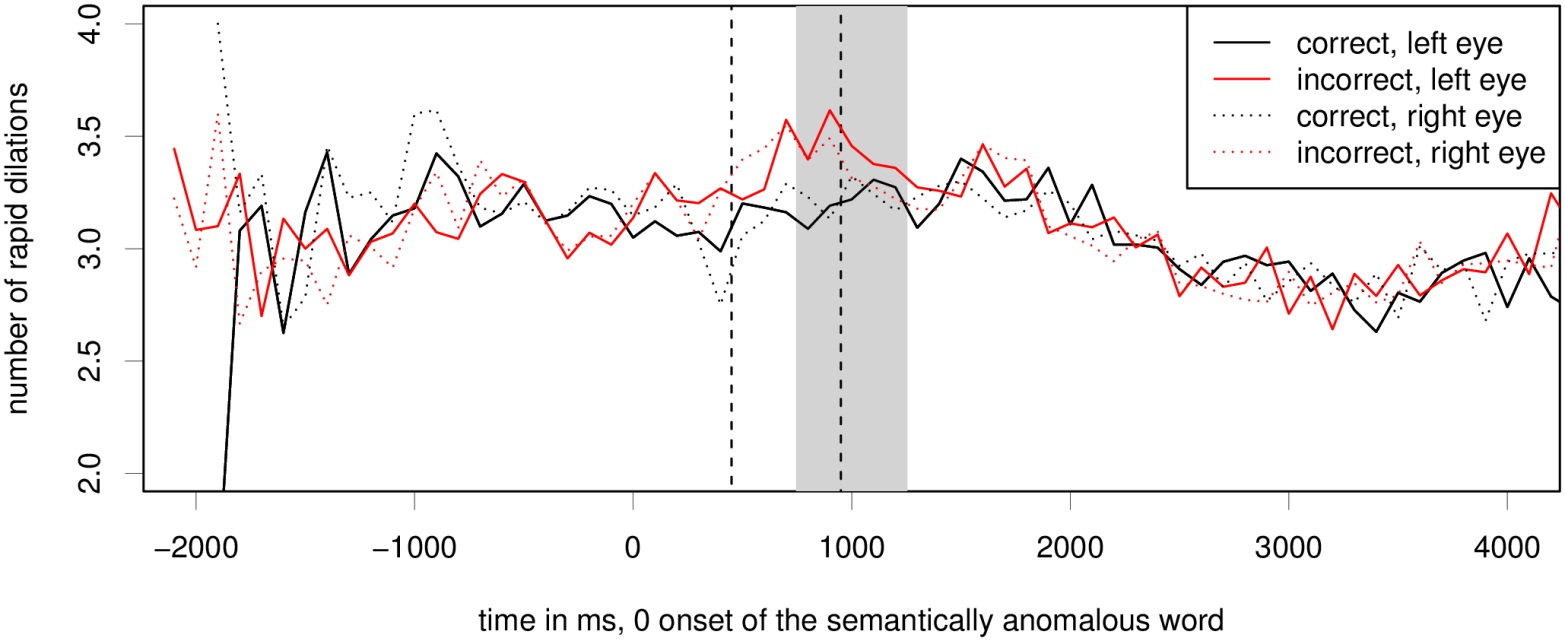
Raw ICA for the dual-task semantic violation experiment. Critical region onset for the experiment is located at word number 0; the grey bar marks the critical region of 500ms, peaking at 1s. Differences between conditions are not statistically significant for this region. However, there is an earlier significant effect for the 500ms time window peaking at 700ms after word onset (indicated here by dashed vertical bars); see Table 6.

Fig 10 shows the average number of rapid dilations for 100ms bins. We can see that the effect by semantic fit condition seems to be about 200–300ms earlier for this experiment than the critical region used for the other experiments.

For this experiment, we find that none of our predictor variables other than steering movement significantly improved model fit for the critical region peaking at 1s post stimulus onset. In particular, there is no significant effect of our semantic manipulation. However, Fig 10 shows a slightly earlier effect for this experiment. Therefore, we also tested the time period peaking at 700ms, which is reported in Table 6. For this earlier time period, we find a significant effect of semantic violation on both eyes. When applying Bonferroni correction for testing this second time interval, the effect is statistically significant at *p* = 0.024. For this time interval, item order, the interaction between item order and semantic condition, steering bar speed and X and Y fixation positions did not significantly improve model fit. We however did find a significant effect of semantic anomaly, as well as a significant negative effect of large detected eye movements along the y axis. The resulting model is significantly better (*χ*^2^ = 5.752, *p* = 0.016) than a model without the main effect of semantic condition.

**Table 6:** Linear mixed effects models for left and right eye in dual task semantic match/mismatch experiment for a 500ms time interval peaking at 700ms post onset of the semantically anomalous word.

	ICA			
	*β*	z-val	p-val	95%CI
(Intercept)	2.728	64.28	<0.00001 ***	[2.646, 2.812]
sem mismatch	0.094	2.51	0.012 *	[0.021, 0.168]
movement Y axis	−0.054	−2.65	0.008 **	[−0.094, −0.014]

RawICA = sem mismatch+ Y move + (sem mismatch| item) +(sem mismatch + Y move| participant)+(1| participant:eye)

In a model for the left eye only and full random effects structure, a main effect of semantic condition significantly improves model fit (*χ*^2^ = 7.29, *p* = 0.0069), and is a significant positive predictor (*β* = 0.0925, *p* = 0.014); the same result also holds for the right eye (*χ*^2^ = 4.709, *p* = 0.03; coefficient for semantic mismatch: *β* = 0.098, *p* = 0.0229).

## Experiment 6: Relative clauses in dual task driving and listening

### Procedure

Procedure was identical to the one described for experiment 4.

### Materials

Materials for this experiment were identical to materials used for experiment 3, and speech-synthesized using the MARY synthesizer, as in experiment 4. The duration of the critical *hat/haben* region in the relative clause stimuli was manipulated by adding some silence after *hat* such that the duration of the critical region was always 650ms long.

### Data Analysis

Data analysis was done in the same way as described for experiments 4 and 5.

### Results

#### Question Answering Accuracies

Question answering accuracies for the dual task relative clause experiment showed a similar pattern as the ones for experiment 4. 86.2% of participants correctly answered “yes” in the subject relative clause condition, and 65.1% of participants correctly answered “yes” in the object relative clause condition.

#### Index of Cognitive Activity

**Fig 11:**
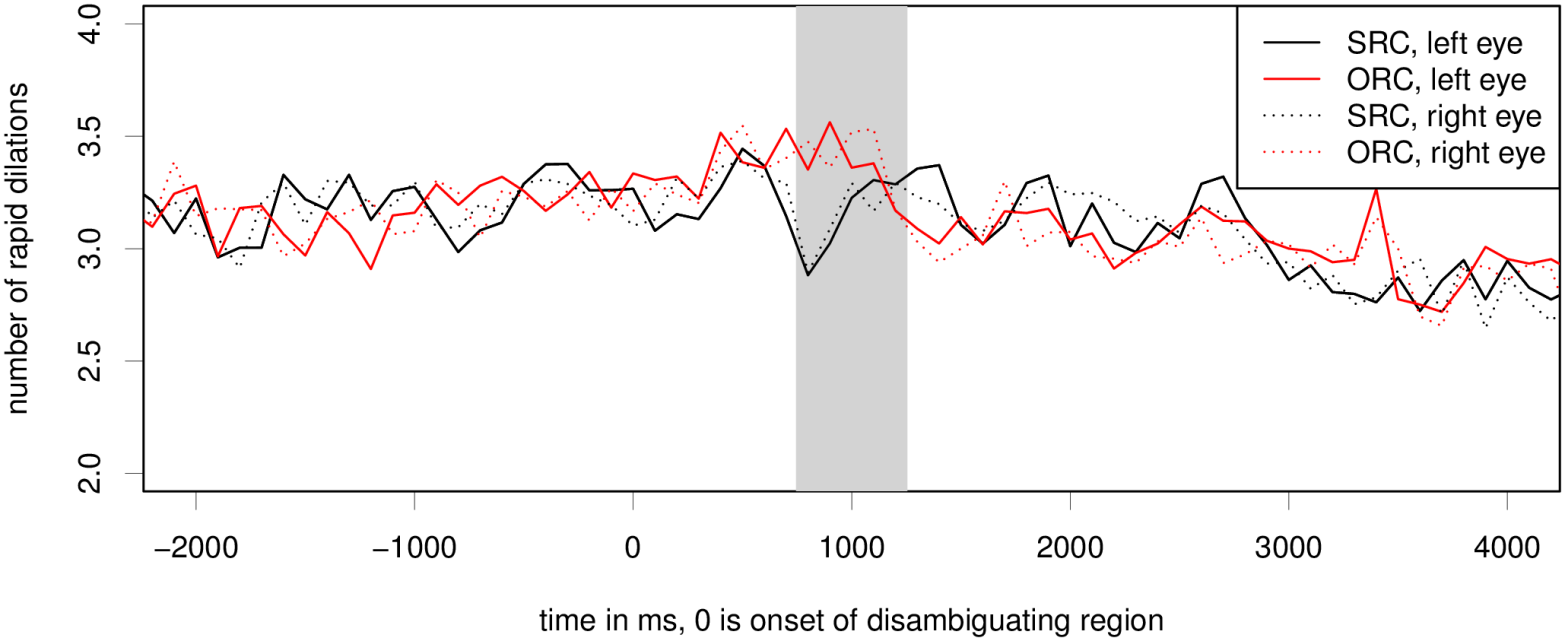
Raw ICA for the dual-task relative clause experiment. Critical region onset for the experiment is located at word number 0; the gray bar marks the hypothesized critical region of 500ms, peaking at 1s. The differences between the conditions are statistically significant at *p* < 0.05 for both eyes; see also Table 7.

Fig 11 shows a clear effect of relative clause type on the number of rapid pupil dilations. There is an increased number of rapid pupil dilations during a period of approximately 400 to 1100ms after onset of the critical region *hat / haben* for the object relative clause condition, while there is a much shorter peak for the subject relative clause condition. Below, we will test for significance of the relative clause effect in the critical region of 750 to 1250ms.

Backward model selection shows that X and Y axis position as well as item order and the interaction of item order with relative clause type are not significant predictors of rapid dilations for either eye. However, including predictors for steering movement as well as relative clause condition significantly improves model fit (left eye: *χ*^2^ = 5.607, *p* = 0.018; right eye:*χ*^2^ = 6.83, *p* = 0.009; both: *χ*^2^ = 5.456, *p* = 0.0195), comparing a model with vs. without a main effect of relative clause condition and full random effects structure. Table 7 shows results for a model including both eyes for the time period of 750–1250ms after onset of the critical region. A model including only the left eye estimates the coefficient for the object relative clause at *β* = 0.069, *p* = 0.0127; for a model including only the right eye, the estimates are *β* = 0.083, *p* = 0.005). All models include random slopes for relative clause condition under participant and item.

**Table 7:** Linear mixed effects models for left and right eye in dual task relative clause experiment.

	ICA			
	*β*	z-val	p-val	95%CI
(Intercept)	2.744	92.26	<0.0001 ***	[2.686, 2.803]
object RC	0.069	2.46	0.014 *	[0.014, 0.125]
steering	0.064	3.65	0.0002 ***	[0.030, 0.099]

RawICA = RC +steering+(RC| item) +(RC + steering| participant) +(1| participant:eye)

### Discussion of Relative Clause Dual Task Experiment

Differences between this experiment and the self-paced reading experiment using the same materials lie in the presentation rate: in the self-paced reading experiment, participants were very slow to press the button to proceed to the next word in the object relative clause condition, while the dual-task experiment here was not self-paced, and also controlled for the exact duration of the critical region by inserting a few ms of pause after *hat*. We can’t exclude that the possibility that the difference between conditions in this experiment is also influenced by the slightly longer prosodic break in the subject relative clause condition (although we would like to point out that there is a clear prosodic boundary at the end of the relative clause in both conditions, and the difference in duration of this prosodic break is too small for listeners to consciously notice it).

A further difference is in the peak of frequent dilations that we saw in the time period of 500–900ms after appearance of the critical word, which we interpreted as a sign of increased attention at the critical region of the relative clause, but which we do not observe in the current experiment. This difference might be due to the dual-task setting, where participants were more distracted and hence did not engage in as much predictive processing or attention to the disambiguating region of the relative clause.

## Overall Discussion for ICA in dual task experiments

The results of the single task experiments 1–3 and the dual task experiments 4–6 are remarkably consistent with one another, even though the modalities for the self-paced reading task and the listening while driving task were very different. The overall time course of the effects was also quite stable: with the exception of the semantic anomaly experiment in the dual task condition, we found a significant difference between linguistic conditions in all experiments for the time period of 750 to 1250ms after critical region onset, a time period that is also consistent with the ICA effect in the driving task (peak of correlation between stimulus and ICA).

When comparing the plotted averages of ICA values, we can, however, also observe small differences in the time course of the different manipulations. Experiments 1, 2, 5 and 6 showed an effect that is slightly earlier than the critical region we tested, while experiment 4 shows the largest difference between conditions slightly later than our critical region. These differences in exact peaks can likely be attributed to the different time courses of word perception and processing during the critical region. We know from EEG that auditory experiments often show slightly earlier effects than written presentation, but our auditory experiments also differ in when exactly the relevant difference between conditions becomes perceptible: in the gender marking experiment, the mismatch is only evident at the end of the word, once it is clear that the word is not a compound but indeed inconsistent with the article; in the semantic anomaly experiment, the inconsistency can likely be perceived before the end of the word, and for the relative clause experiment, the words *hat* and *haben* also only differ at the word offset. This pattern is consistent with the time course observed in our studies, where the effect is earliest for the semantic experiment (expt 5) and latest for the grammatical gender experiment (expt 4).

## Experiment 7: ICA in Visual World Experiment

Our final experiment tests whether the ICA can serve as a useful measure in experiments using the visual world paradigm. The visual world paradigm is popular in language research, because people’s locus of visual attention can offer us insight into the language interpretation and time course of processing. However, the visual world paradigm does not allow us to get any insight into how *difficult* it is for a subject to get to a specific interpretation. Here, we want to explore whether the ICA is sufficiently robust to changing fixation positions on the screen in order to allow us to use it to asses cognitive load in addition to visual attention when using the visual world paradigm.

The visual world experiment reported here contains a more subtle linguistic difference than the earlier experiments: it tests whether a concessive discourse connector is more difficult to process than a causal one, because it requires a reconstruction of expectations to an event that contrasts with the event that would be expected by default (or following the causal connector). Evidence from an EEG experiment using similar materials in a reading experiment revealed a P600 effect on the connective [37, 38].

### Participants

We collected data from 24 participants. Data from one of the participants was not recorded correctly, so that we had to discard the data before analysis. This left us with 23 participants, thereof 14 females, 22 right eye dominant, 17 right-handed, age 19–30 years old, mean age 22.7 years. All participants had normal or corrected to normal vision and were native speakers of German.

### Materials

We constructed 20 items and 40 fillers, each consisting of three spoken sentences in German, and a static scene (see Example (4); the materials are the same as for the experiment reported in [39]; see also S2 Fig provided with this publication and in the data repository http://dx.doi.org/10.7910/DVN/JDEVQV). The sentences were spoken by a native speaker of German. As fillers, we used texts and pictures which were designed such that they would seem to the participants to have a similar structure, but which did not include any causal or concessive relations.

(4) *Marc denkt über einen kleinen [Snack nach. Er hat gerade Lust, etwas]*_*topic*_ *[Süßes / Salziges zu essen]*_*category*_. *[**Daher** / **Dennoch** holt er sich]*_*connector*_ *[aus der Küche]*_*extended*_ *[die appetitliche / den appetitlichen]*_*pretarget*_ *[Waffel / Kuchen / Brezel / Käse]*_*target*_.
  “Marc fancies a [snack. He feels like having something]_*topic*_ [sweet]_*category*_. [**Therefore** / **Nevertheless**, he gets]_*connector*_ [from the kitchen]_*extended*_ [the_[*fem*]/[*masc*]_ delicious_[*fem*]/[*masc*]_]_*pretarget*_ [waffle / cake / pretzel / cheese]_*target*_.”

### Procedure

The spoken stimuli were played while participants could freely look at a picture on the screen containing target objects as well as distractors. Some of the items were followed by comprehension questions which tested the interpretation of the causal / concessive connector. These questions were asked in order to make sure that subjects were reading for comprehension, and to assess whether they interpreted the stimuli correctly. The order of items and fillers was randomized.

### Data Analysis

For our regression models, we again excluded all data points where the pupil size estimate was smaller than 2.5 standard deviations from the average pupil size of that participant, as in all other experiments. To make sure that effects measured in the ICA or in pupil size are not simply due to fixation position on the screen, we also entered the fixation coordinates and changes in fixation position during the critical region due to eye movements as predictors in our regression models. All reported models again include random slopes for connector type under item and participant.

### Results

**Fig 12:**
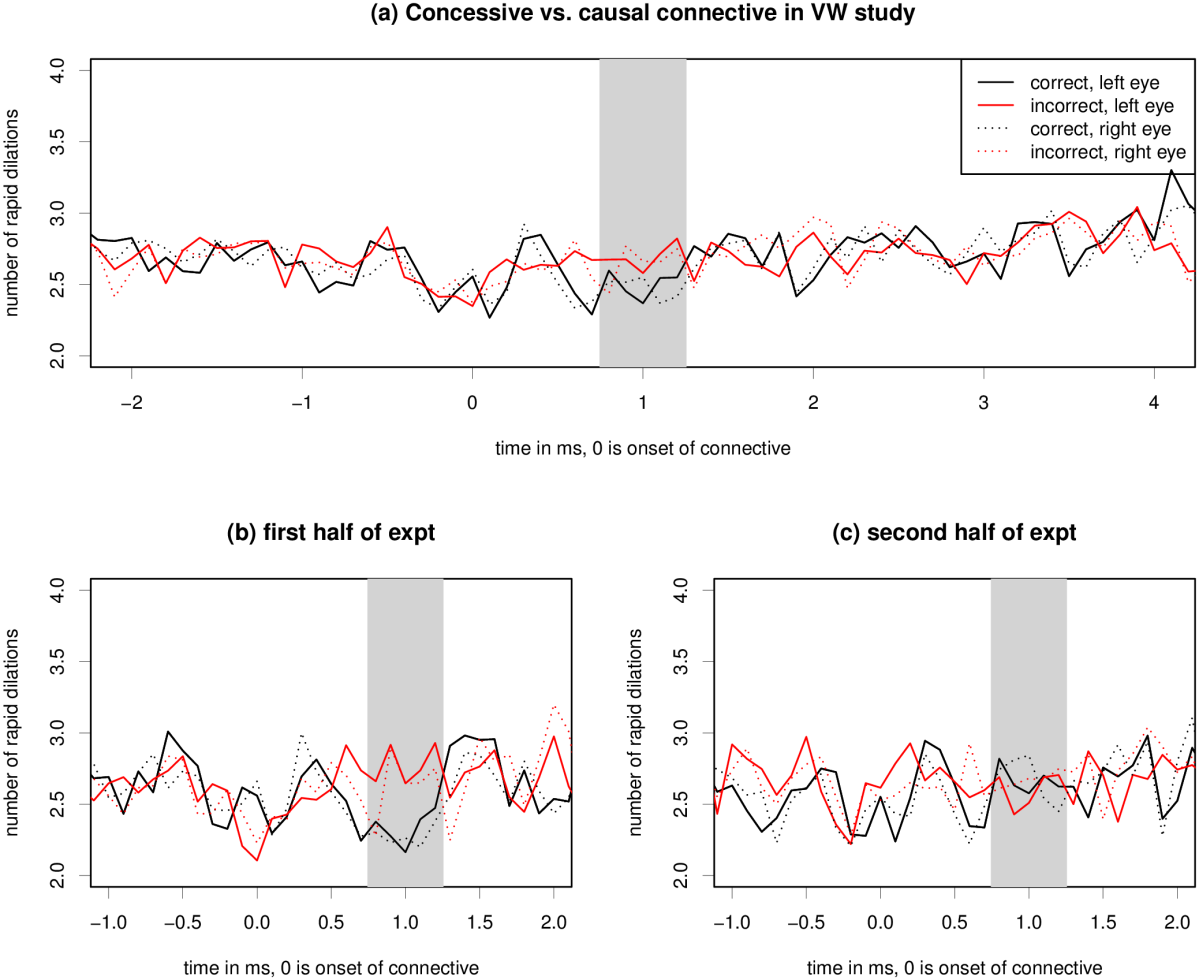
Raw ICA for the connective region of the visual world experiment. Critical region onset for the experiment is located at word number 0. The grey bar marks the hypothesized critical region of 500ms, peaking at 1s. See also Tables 8 and 9.

Fig 12 shows that there are more rapid pupil dilations during the period of 600 to 1200ms after onset of the connective. The effect looks smaller than for some of the other experiments which much more strongly violated linguistic or world knowledge, so a smaller effect size is not surprising for the discourse connective visual world study.

The visual world experiment necessarily contains fixations to all parts of the screen (as this is part of the experimental design in this experiment). Data analysis confirms that fixation positions are relatively evenly distributed across the screen, unlike the earlier experiments reported in this article, where most of the looks were to the center of the screen. Using generalized additive models that allow for smooth functions for X and Y fixation positions, we find that the optimal fit is linear. Closer inspection of the data revealed that the effect of fixation position on the ICA was subject to some variance across participants (for the majority, there was a small linear effect while some showed a slight effect for increased eccentricity from the center of screen). Importantly, the effects were independent of the experimental manipulation: regression models with and without fixation position as a linear, quadratic, or non-parametric predictor all yield equivalent results with respect to the estimate of our linguistic predictor for connector type. We also included eye movement distance in X or Y direction (i.e., quantifying the amount of change in fixation position) during the critical region as a linear predictor in the model. For all models, we performed backward model fitting, as in experiments 1–6.

We found that item order as well as the interaction of item order and connective type significantly improved model fit. Furthermore, fixation position with respect to the Y axis and the size of vertical eye movements were significant (negative) predictors of the ICA. We hypothesize that this might be also due to small amounts of drift with our head-mounted eye-tracker. X axis fixation position and eye movements along the X axis were not predictive of the ICA.

On the full data, we did not find a significant main effect of connective type when random slopes under item and participant were included for this predictor, see also Table 8. The significant negative interaction between connective type and item order however indicates that there was an effect of connective type in the beginning of the experiment, which then decreased as the experiment proceeded. In order to test this hypothesis, we ran a regression model on the first half of the experiment only, see also Fig 12(b). For the first half of the experiment, we found that the ICA was significantly higher for concessive connectives compared to causal connectives, see Table 9. Item order and the interaction of item order and connective type were not predictive in this model.

**Table 8:** Linear mixed effects models for left and right eye in dual task gender match/mismatch.

ICA: full experiment				
	*β*	z-val	p-val	95%CI
(Intercept)	2.489	47.26	<0.0001 ***	[2.385, 2.592]
concessive	0.077	1.18	0.2377	[−0.051, 0.206]
item order	0.079	2.48	0.0133 *	[0.016, 0.143]
Y axis movement	−0.057	−5.15	2.65e-07 ***	[−0.079, −0.036]
Y axis fixation pos	−0.092	−8.11	5.06e-16 ***	[−0.114, −0.069]
concessive:item order	−0.095	−2.15	0.0319 *	[−0.183, −0.008]

RawICA = concessive * item order + Y move + Y pos+(concessive | item) + (concessive * item order | participant) + (1 | participant:eye)

**Table 9:** Linear mixed effects models for left and right eye in dual task gender match/mismatch; first half of experiment.

ICA: first half of experiment				
	*β*	z-val	p-val	95%CI
(Intercept)	2.398	31.89	<0.0001 ***	[2.251, 2.545]
concessive	0.188	2.20	0.028 *	[0.020, 0.357]
Y axis movement	−0.092	−5.57	2.50e-08 ***	[−0.124, −0.059]
Y axis fixation pos	−0.089	−5.47	4.54e-08 ***	[−0.122, −0.057]

RawICA = concessive + Y move + Y pos + (concessive |item) +(concessive | participant) + (1 | participant:eye)

Separate analyses for each of the eyes are consistent with this result: for the first half of the experiment, we find a significant effect of connective type on the left eye: *β* = 0.19, *p* = 0.028 and on the right eye: *β* = 0.17, *p* = 0.035 (both models include random slopes for connector type under both item and participant).

### Discussion for ICA in visual world experiment

Our visual world experiment provides first evidence that the ICA might constitute a useful measure to simultaneously assess visual attention and processing difficulty in psycholinguistic experiments using the visual world paradigm. Our results (significantly higher ICA on the concessive connector compared to the causal one) are consistent with earlier findings in a closely related experiment using EEG, in which a P600 effect was found in a similar time window as the ICA effect [37]. However, these results were only apparent in the first half of the experiment: we interpret this finding in terms of a learning effect such that the difference in ICA effect between the connectives was reduced during the experiment and disappeared by the end of the experiment. Learning effects are well-known to affect psycholinguistic experiments, and in particular affect those conditions more which are less likely a-priori [40]. In the case of our study, this means that the concessive connective initially leads to higher processing difficulty as compared to the causal connective, but as the experiment proceeds and participants are exposed to many concessives, they potentially begin to expect concessives to appear, and as a result experience less processing difficulty when encountering a concessive in the second half of the experiment.

This experiment elicited considerably more eye movements than the other experiments presented in this article. We therefore wondered whether the ICA estimates would be affected by eye-movements, and if so, whether eye-movements potentially would introduce a level of noise that would mask the effect of our manipulation. We found that effects of fixation position were small and that including fixation position as a predictor in our models did not affect estimates of the linguistic manipulation. We therefore conclude that the ICA constitutes a promising measure to assess visual attention and processing difficulty simultaneously.

## Conclusions

The experiments presented here provide strong evidence that the ICA is sensitive to linguistic manipulations, showing significant effects in the expected direction on both eyes during the pre-defined time window of 750–1250ms post stimulus onset in all experiments except one (experiment 5), for which we found a significant effect 250ms earlier. In two of the experiments we also observed learning effects: in experiment 7 (visual world) and experiment 3 (relative clauses in self-paced reading), we found that the effect of the experimental manipulation was statistically significant in the first half of the experiment, but disappeared during the second half of the experiment.

Due to the ICA being a highly dynamic measure (the auto-correlation is very low), we had hypothesized that it would be possible to disentangle the effects of overlapping stimuli. The single task driving analysis presented as part of experiment 4 showed that the ICA reflects difficulty of the steering task. We were then able to show in experiments 4–6 that both effects can be accounted for separately in a linear mixed effects model. Effects of the linguistic manipulation were detectable in the dual task setting, with effect sizes similar to the single task experiments 1–3.

These results are very encouraging for the future use of the ICA as a measure for assessing linguistically induced cognitive load. We see large application potential for this measure in particular in experiments where traditional eye-movement measures are not applicable, for example, in studies using auditory stimuli, or within the visual world paradigm to assess visual attention and cognitive processing effort simultaneously. Our study reported in this article provides evidence that the ICA is applicable as a measure of processing difficulty that does not first have to account for artefacts introduced through eye-movements or changes in luminosity of the screen (e.g., when fixating at parts of the screen with lighter or darker objects, or a background of different colour, as such changes affect overall pupil size). A further application field is in dual task settings, as demonstrated here in experiments 4–6, where physical movement is required by one of the tasks, making the use of EEG difficult.

In the introduction, we pointed out the possible relation between the ICA and the locus caeruleus area of the brain stem, as well as the possible role of the LC/NE system in the P3b/P600. The P600-as-P3b-LC/NE-hypothesis predicts that the P600 (and other effects that are related to activity of the LC/NE system) should be reaction-time aligned (as opposed to being stimulus-aligned). While our experimental settings did not involve reaction time experiments in our language tasks and our experimental data hence does not allow us to test whether the ICA is aligned with linguistic response times, the single task driving experiment (presented as part of Experiment 4) does allow us to compare the ICA to reaction times (steering as a reaction to the movement of the reference bar). As reported in this article, we found that the ICA is more strongly correlated in time with our subjects’ steering movements than with the stimulus (movement of the reference bar). This observation is fully in line with our suggested interpretation of the ICA. A more direct experiment showing this for the linguistic item should be conducted in the future. Furthermore, findings in the literature about the LC region have related activity in the LC region to attention and salience. Linguistic errors or incoherences as presented here as well as the difficult relative clauses are highly salient (as also evidenced by the reading times in the single task reading experiments), i.e., they draw attention. The observed increased levels of ICA in the critical regions of the violation (remember in particular the peak in rapid dilations in the critical region of both conditions in experiment 3) are consistent with such an attentional mechanism as well as with the observed improvement of performance during the course of the single task relative clause experiment.

Hence, there exists a potentially very interesting relation between the ICA index and more established measures such as the P600 in ERP studies.

## Supporting Information

S1 Text Experimental Materials for Experiments 1 to 6 (grammatical gender mismatch, semantic fit, relative clauses). (TXT)

S1 Fig Screenshot of the ConTRe driving task scene (ConTRe steering only), used in Experiments 4–6. For our experiments, we removed the traffic light which is visible on top of the yellow bar. (PNG)

S2 Text Experimental materials (language stimuli) for experiment 7 (causal vs. concessive discourse connectors). (TXT)

S2 Fig Experimental visual stimulus for first item, for full bitmaps and audio files see data repository. (BMP)

## References

[1] Hess EH, Polt JM. Pupil size as related to interest value of visual stimuli. Science. 1960;. 10.1126/science.132.3423.349 14401489

[2] Hess EH, Polt JM. Pupil size in relation to mental activity during simple problem-solving. Science. 1964;. 10.1126/science.143.3611.1190 17833905

[3] Kahneman D, Beatty J. Pupil diameter and load on memory. Science. 1966;. 10.1126/science.154.3756.1583 5924930

[4] Beatty J. Task-evoked pupillary responses, processing load, and the structure of processing resources. Psychological bulletin. 1982;91(2):276 10.1037/0033-2909.91.2.276 7071262

[5] Schluroff M. Pupil responses to grammatical complexity of sentences. Brain and language. 1982;17(1):133–145. 10.1016/0093-934X(82)90010-4 7139265

[6] Just MA, Carpenter PA. The intensity dimension of thought: pupillometric indices of sentence processing. Canadian journal of experimental psychology. 1993;47(2):310–339. 10.1037/h0078820 8364533

[7] Hyönä J, Tommola J, Alaja AM. Pupil dilation as a measure of processing load in simultaneous interpretation and other language tasks. The Quarterly Journal of Experimental Psychology. 1995;48(3):598–612. 10.1080/14640749508401407 7568993

[8] Zellin M, Pannekamp A, Toepel U, der Meer E. In the eye of the listener: Pupil dilation elucidates discourse processing. Int Journal of Psychophysiology. 2011;. 10.1016/j.ijpsycho.2011.05.009 21679730

[9] Frank SL, Thompson RL. Early effects of word surprisal on pupil size during reading. In: Proc. 34th Annu. Conf. Cognitive Science Society (eds N. Miyake, D. Peebles & RP Cooper); 2012. p. 1554–1559.

[10] Engelhardt PE, Ferreira F, Patsenko EG. Pupillometry reveals processing load during spoken language comprehension. Quarterly journal of experimental psychology. 2010;63:639–645. 10.1080/17470210903469864 20017058

[11] Tromp J, Hagoort P, Meyer AS. Pupillometry Reveals Increased Pupil Size During Indirect Request Comprehension. The Quarterly Journal of Experimental Psychology. 2015;p. 1–16. 10.1080/17470218.2015.1065282 26110545

[12] Marshall SP. Method and apparatus for eye tracking and monitoring pupil dilation to evaluate cognitive activity; 2000. US Patent 6,090,051.

[13] Marshall SP. The index of cognitive activity: Measuring cognitive workload. In: proc. th conference on Human factors and power plants. IEEE; 2002. p. 7–5.

[14] Marshall SP. Identifying cognitive state from eye metrics. Aviation, space, and environmental medicine. 2007;78(Supplement 1):B165–B175. 17547317

[15] Demberg V. Pupillometry: the Index of Cognitive Activity in a dual-task study. In: Proceedings of the 35th Annual Meeting of the Cognitive Science Society (CogSci-13); 2013. p. 2154–2159.

[16] Aston-Jones G, Cohen JD. An integrative theory of locus coeruleus norepinephrine function: adaptive gain and optimal performance. Annual review of neuroscience. 2005;28:403–450. 10.1146/annurev.neuro.28.061604.135709 16022602

[17] Laeng B, Sirois S, Gredebäck G. Pupillometry: a window to the preconscious? Perspectives on psychological science. 2012;7(1):18–27. 26168419 10.1177/1745691611427305

[18] Sara SJ. The locus coeruleus and noradrenergic modulation of cognition. Nature reviews neuroscience. 2009;10(3):211–223. 10.1038/nrn2573 19190638

[19] Berridge CW, Waterhouse BD. The locus coeruleus—noradrenergic system: modulation of behavioral state and state-dependent cognitive processes. Brain Research Reviews. 2003;42(1):33–84. 10.1016/S0165-0173(03)00143-7 12668290

[20] Samuels E, Szabadi E. Functional neuroanatomy of the noradrenergic locus coeruleus: its roles in the regulation of arousal and autonomic function part I: principles of functional organisation. Current neuropharmacology. 2008;6(3):235 10.2174/157015908785777229 19506723 PMC2687936

[21] Nieuwenhuis S, Aston-Jones G, Cohen JD. Decision making, the P3, and the locus coeruleus—norepinephrine system. Psychological bulletin. 2005;131(4):510 10.1037/0033-2909.131.4.510 16060800

[22] Coulson S, King JW, Kutas M. Expect the unexpected: Event-related brain response to morphosyntactic violations. Language and cognitive processes. 1998;13(1):21–58. 10.1080/016909698386582

[23] Sassenhagen J, Schlesewsky M, Bornkessel-Schlesewsky I. The P600-as-P3 hypothesis revisited: Single-trial analyses reveal that the late EEG positivity following linguistically deviant material is reaction time aligned. Brain and language. 2014;137:29–39. 10.1016/j.bandl.2014.07.010 25151545

[24] Makeig S, Delorme A, Westerfield M, Jung TP, Townsend J, Courchesne E, et al Electroencephalographic brain dynamics following manually responded visual targets. PLoS biology. 2004;2(6):e176 10.1371/journal.pbio.0020176 15208723 PMC423146

[25] Schwalm M. Pupillometrie als Methode zur Erfassung mentaler Beanspruchungen im automotiven Kontext. Universitätsbibliothek der Universität des Saarlandes; 2008.

[26] Schwalm M, Keinath A, Zimmer HD. Pupillometry as a method for measuring mental workload within a simulated driving task. Human Factors for assistance and automation. 2008;p. 1–13.

[27] Loewenfeld IE, Lowenstein O. The pupil: Anatomy, physiology, and clinical applications. vol. 2 Wiley-Blackwell; 1993.

[28] Klingner J, Kumar R, Hanrahan P. Measuring the task-evoked pupillary response with a remote eye tracker. In: Proceedings of the 2008 symposium on Eye tracking research & applications. ACM; 2008. p. 69–72.

[29] Bartels M, Marshall SP. Measuring cognitive workload across different eye tracking hardware platforms. In: Proceedings of the symposium on eye tracking research and applications. ACM; 2012. p. 161–164.

[30] Bates D, Maechler M, Bolker B, Walker S. Linear mixed-effects models using Eigen and S4. R package version 1.0–5; 2013.

[31] Wood S, Scheipl F. gamm4: Generalized additive mixed models using mgcv and lme 4; 2014. R package version 0.2–3. Available from: http://CRAN.R-project.org/package=gamm4.

[32] Bader M, Meng M. Subject-object ambiguities in German embedded clauses: An across-the-board comparison. Journal of Psycholinguistic Research. 1999;28(2):121–143. 10.1023/A:1023206208142

[33] Mahr A, Feld M, Moniri MM, Math R. The ConTRe (Continuous Tracking and Reaction) Task: A Flexible Approach for Assessing Driver Cognitive Workload with High Sensitivity. In: Automotive user interfaces and interactive vehicular applications; 2012 p. 88–91.

[34] Viviani P, Mounoud P. Perceptuomotor compatibility in pursuit tracking of two-dimensional movements. Journal of motor behavior. 1990;22(3):407–443. 10.1080/00222895.1990.10735521 15117667

[35] Schröder M, Trouvain J. The German text-to-speech synthesis system MARY: A tool for research, development and teaching. International Journal of Speech Technology. 2003;6(4):365–377. 10.1023/A:1025708916924

[36] Wood SN. mgcv: GAMs and generalized ridge regression for R. R news. 2001;1(2):20–25.

[37] Drenhaus H, Demberg V, Koehne J, Delogu F. Incremental and predictive discourse processing based on causal and concessive discourse markers: ERP studies on German and English. In: Proceedings of the 36th Annual Meeting of the Cognitive Science Society (CogSci-14); 2014. p. 403–408.

[38] Xiang M, Kuperberg G. Reversing Expectations: The Use of Concessive Connectives during Discourse Comprehension. Language, Cognition and Neuroscience. 2014;. 10.1080/23273798.2014.995679 PMC4405243 25914891

[39] Köhne J, Demberg V. The Time-course of Processing Discourse Connectives. In: Knauff M, Pauen M, Sebanz N, Wachsmuth I, editors. Proceedings of the 35th Annual Meeting of the Cognitive Science Society (CogSci-13); 2013. p. 2760–2765.

[40] Fine AB, Jaeger TF, Farmer TA, Qian T. Rapid Expectation Adaptation during Syntactic Comprehension. PLoS ONE. 2013;8:77661 10.1371/journal.pone.0077661 PMC3813674 24204909

