# Supplementary figures and images for: The Frequency of Rapid Pupil Dilations as a Measure of Linguistic Processing Difficulty
Source: PLoS One. 2016 Jan 22;11(1):e0146194. doi: 10.1371/journal.pone.0146194 (PMC4723154; doi:10.1371/journal.pone.0146194)

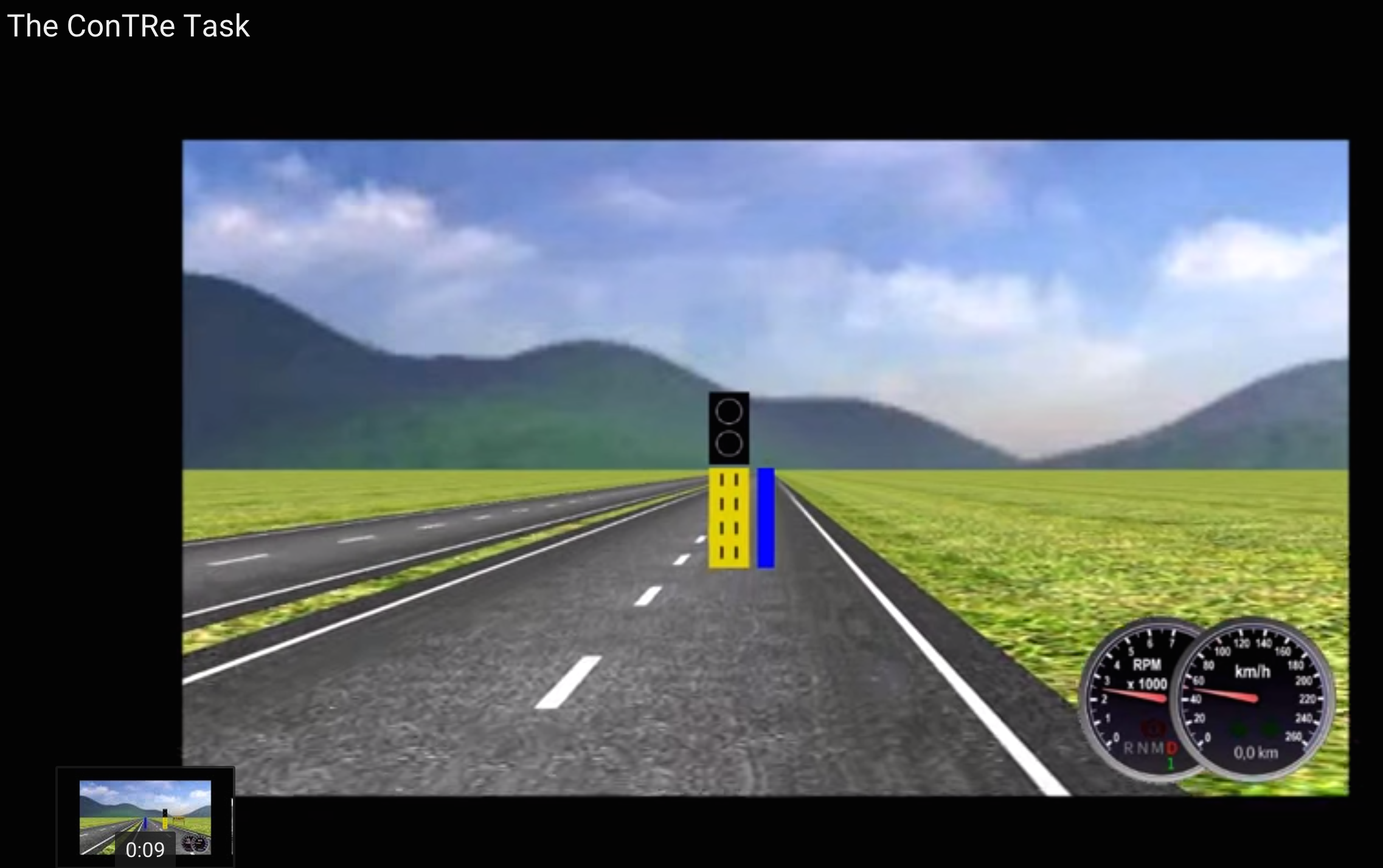

Supplement: S1 Fig — (PNG) [file pone.0146194.s002.png]

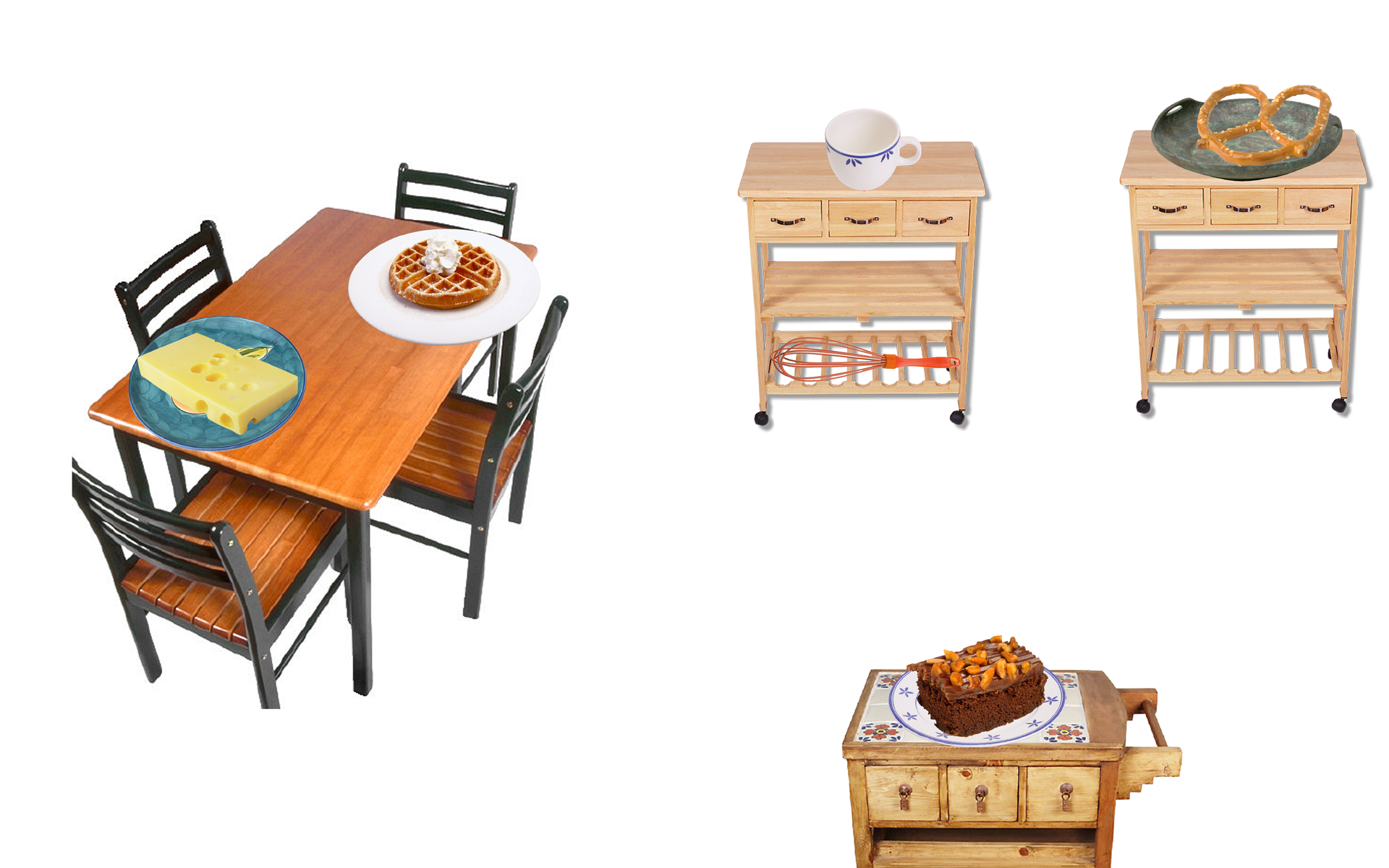

Supplement: S2 Fig — (BMP) [file pone.0146194.s004.bmp]
